# Otitis media: a genome-wide linkage scan with evidence of susceptibility loci within the 17q12 and 10q22.3 regions

**DOI:** 10.1186/1471-2350-10-85

**Published:** 2009-09-03

**Authors:** Margaretha L Casselbrant, Ellen M Mandel, Jeesun Jung, Robert E Ferrell, Kathleen Tekely, Jin P Szatkiewicz, Amrita Ray, Daniel E Weeks

**Affiliations:** 1 Division of Pediatric Otolaryngology, Children's Hospital of Pittsburgh of University of Pittsburgh Medical Center, Pittsburgh, PA, 15201, USA; 2 Department of Otolaryngology, University of Pittsburgh, School of Medicine, Pittsburgh, PA, 15213, USA; 3 Department of Human Genetics, Graduate School of Public Health, University of Pittsburgh, Pittsburgh, PA, 15261, USA; 4 Department of Biostatistics, Graduate School of Public Health, University of Pittsburgh, Pittsburgh, PA, 15261, USA

## Abstract

**Background:**

Otitis media (OM) is a common worldwide pediatric health care problem that is known to be influenced by genetics. The objective of our study was to use linkage analysis to map possible OM susceptibility genes.

**Methods:**

Using a stringent diagnostic model in which only those who underwent tympanostomy tube insertion at least once for recurrent/persistent OM are considered affected, we have carried out a genome-wide linkage scan using the 10K Affymetrix SNP panel. We genotyped 403 Caucasian families containing 1,431 genotyped individuals and 377 genotyped affected sib pairs, and 26 African American families containing 75 genotyped individuals and 27 genotyped affected sib pairs. After careful quality control, non-parametric linkage analysis was carried out using 8,802 SNPs.

**Results:**

In the Caucasian-only data set, our most significant linkage peak is on chromosome 17q12 at rs226088 with a p-value of 0.00007. Other peaks of potential interest are on 10q22.3 (0.00181 at rs1878001), 7q33 (0.00105 at rs958408), 6p25.1 (0.00261 at rs554653), and 4p15.2 (0.00301 at rs2133507). In the combined Caucasian and African American dataset, the 10q22.3 peak becomes more significant, with a minimal p-value of 0.00026 at rs719871. Family-based association testing reveals signals near previously implicated genes: 513 kb from *SFTPA2 *(10q22.3), 48 kb from *IFNG *(12q14), and 870 kb from *TNF *(6p21.3).

**Conclusion:**

Our scan does not provide evidence for linkage in the previously reported regions of 10q26.3 and 19q13.43. Our best-supported linkage regions may contain susceptibility genes that influence the risk for recurrent/persistent OM. Plausible candidates in 17q12 include *AP2B1*, *CCL5*, and a cluster of other CCL genes, and in 10q22.3, *SFTPA2*.

## Background

Otitis media (OM [MIM 166760]) is a worldwide pediatric health care problem. Nearly all children experience at least one episode of acute OM (AOM). Otitis media, particularly in children who experience recurrent/persistent disease, in addition to the short- and long-term effect on the child places considerable financial burden on the families (parental time, transportation, medications) and on the health-care system (clinic visits, medical and surgical therapies, complications). In 1996, Gates [1] estimated the costs for AOM and chronic otitis media with effusion (COME) in the U.S. at approximately 3 billion and 2 billion dollars, respectively. Today the annual cost can be expected to be much higher [2]. In addition to the financial burden, the psychosocial impact of OM on the child and the family is tremendous, but has not been well studied.

Epidemiologic studies using various methodologies have demonstrated evidence that genetics plays an important role in recurrent and persistent OM: population studies [3-10]; adoption study [11]; familial aggregation studies [12-16]; and twin and triplet studies. The estimated otitis media heritability in the retrospective twin study by Kvaerner et al [17] was 0.74 in females and 0.45 in males, and in the prospective twin and triplet study of children followed from birth to 2 years of age by Casselbrant et al [18] the heritability of time with middle ear effusion was 0.79 in females and 0.64 in males. Similarly, a prospective study of a community sample of twins estimated heritability of total OM symptom scores as 0.49 to 0.71, depending on age [19].

While there is substantial evidence that genetics plays a role in OM, there has been only one genome-wide linkage scan to date by Daly et al [20] that provided evidence of linkage of COME and recurrent OM (ROM) to 10q26.3 and to 19q13.43. Subsequent multipoint linkage analysis of both regions further strengthened the evidence of linkage [21].

Otitis media is a complex disease with contributions from immunocompetence, inflammatory regulation and craniofacial abnormalities that could lead to Eustachian tube dysfunction; therefore, the underlying genetic determinants are likely to be complex and involve several loci. Ilia et al [22] in a recent review describe how case-control studies and animal studies have implicated genes involved in non-specific immunity (epithelial barriers and mucins), innate immunity (toll-like receptors, mannose-binding lectin, surfactant proteins, cytokines), and adaptive immunity (Fc-γ receptors, immunoglobins).

Our present study aims to localize genes that contribute to susceptibility to recurrent/persistent middle ear disease by carrying out a genome-wide screen for linkage using families containing at least two affected siblings. The identification of susceptibility genes may enable us to better understand the pathogenesis of OM leading to development of better and more innovative methods for prevention and treatment, which is especially important in this era of multi-drug resistant bacteria.

## Methods

### Population, Enrollment and Assessment

Full siblings, two or more, who had a history of tympanostomy tube insertion due to a significant history of OM, their parent(s) and other full sibling(s) with no history of tympanostomy tube insertion were eligible for the study. There was no exclusion of subjects based on gender or race. The study was explained in detail to the parent(s) and children, and informed consent was obtained prior to enrollment. This study was approved by the Human Rights Committee of the Children's Hospital of Pittsburgh (CHP) and by the University of Pittsburgh Institutional Review Board.

The study required only one visit to the Ear, Nose and Throat (ENT) Research Center at CHP. A detailed history regarding recurrent/persistent OM was obtained for each enrolled family member, as well as history regarding risk factors such as breast-feeding, day care attendance, siblings, and exposure to smoking for the enrolled children. Medical records of enrolled children were obtained for review whenever possible. When feasible, children and parent(s) had an ear examination using pneumatic otoscopy by the study physicians (MC and EM) who are validated otoscopists [23]. Tympanograms were obtained using a GSI-38 middle ear analyzer (Lucas-Grason-Stadler, INC). Children were excluded for the following reasons: major congenital malformations, medical conditions with a predisposition for OM (e.g. cleft palate, Down syndrome, or other craniofacial malformations), cared for in the Intensive Care Unit as a neonate; been on assisted ventilation, or known sensorineural hearing loss. The subjects were selected from patients who presented to the ENT Research Center from several sources: the ENT Clinic at the CHP and satellite clinics, subjects who were in other studies at the ENT Research Center, or from physician- or self-referral.

### Phenotype definition

In order to assure a history of significant ear disease, two or more full siblings who both or all had undergone tympanostomy tube insertion were enrolled. While still recording each subject's medical history, the need for tympanostomy tube insertion established that the subject's history of middle ear disease was truly significant, resulting in the need for a surgical procedure. A subject was only considered "affected" if he/she had undergone tympanostomy tube insertion at least once for recurrent/persistent OM, while a subject was considered "unaffected" if he/she had never had tympanostomy tubes and had no known history of recurrent/persistent OM. The remaining subjects were considered as having "unknown" disease status.

### DNA isolation and genotyping

EDTA anticoagulated whole blood (5 cc if less than 5 years old; 5-10 cc if 5 years or older; 10-20 cc from parents) was collected from an arm vein in the Pediatric General Clinical Research Center or in the ENT Research Center or in satellite clinics and transferred to the Human Genetics Laboratory within 48 hours. High molecular weight DNA was isolated by the method of Miller et al [24]. Following re-suspension, the DNA was quantitated by fluorometry, diluted to 50 ng/μl, arrayed in a standard 96 well format and stored at -80°C.

Genotyping was carried out using Affymetrix's GeneChip^® ^Human Mapping 10K Array in two different versions: 1) the original GeneChip^® ^Human Mapping 10K Array Xba 131 with 11,560 SNPs with a mean intermarker distance of 210 kb and an average heterozygosity of 0.37, and 2) the newer GeneChip^® ^Human Mapping 10K Array Xba 142 2.0 with 10,204 SNPs with a mean intermarker distance of 258 kb and an average heterozygosity of 0.38. These two chips are very similar to each other: the newer SNP chip was created by removing 1,400 SNPs from the original SNP list and adding approximately 70 new SNPs. Samples were processed according to the GeneChip Mapping Assay Manual (Affymetrix) hybridized at 48°C for 16 hours in an Affymetrix GeneChip Hybridization Oven. After 16 hours the probe arrays were washed and stained according to the GeneChip Mapping Assay Manual using the DNAARRAY_WS2 protocol on the Affymetrix Fluidics Station 400. Arrays were scanned once with the Agilent GeneArray 2500 scanner and analyzed with Affymetrix GeneChip DNA Analysis Software (GDAS) to generate genotype calls for each of the SNP probes on the array. In this study we used the older Xba 131 SNP chip to genotype 515 people, and the newer Xba 142 2.0 SNP chip to genotype 1,216 people.

### Genetic maps

Starting with the available annotation from Affymetrix, we carefully updated it, as a fair number of SNPs are actually bad, hybridizing to multiple positions in the genome. To detect these, we took the primer sequence for each SNP and used BLAST to find its position(s) in the genome, carefully identifying poor multi-hit SNPs, as well as verifying the current best physical position for each good SNP in the data set. After careful resolution of reference sequence (rs) numbers, current physical map positions were retrieved from Ensembl and used to linearly interpolate genetic map positions based on the Rutgers Combined Linkage-Physical Map [25].

### Quality Control

To verify the stated pedigree structures, we carried out relationship testing using a subset of 1,534 well-typed informative autosomal SNPs (with MAF > = 0.40 and > = 99% genotyping rate) and then made a number of changes to the structures to minimize the number of relationship errors while keeping as many people as possible.

PREST identified 73 within-family pairs with p-values < 0.001 [26,27]. Similarly, RELPAIR examined 1,287,210 genotyped relative pairs, and identified 70 within-family pairs whose inferred relationship was > 1,000 times more likely than the putative relationship [28,29]. Checking for relationships between putative families with RELPAIR revealed two families that had entered the study twice.

Gender checking based on PLINK's algorithm led to the removal of genotypes for 6 people because of unresolved gender errors [30]. We replaced the stated gender with the inferred gender in 4 cases where such a change was symmetric in terms of relationship and OM affection status, and so would not alter the linkage results.

A number of conservative changes were made in response to the relationship testing results, including the following: To resolve the 10 putative monozygotic (MZ) twin pairs identified, from the larger families, we removed the least typed member of an MZ pair 6 times, and we completely removed 2 families that each contained only a single inferred MZ pair. To resolve putative half-sib (HS) families, we removed 6 families containing only a single inferred HS pair, and from larger families, we removed a single person to eliminate the putative HS pair 4 times. To resolve the two families that had participated twice in our study, in both cases, only the more completely typed family was kept.

After resolving the relationship errors, we then removed all genotypes for the 250 individuals with a per person genotyping success rate less than 90%.

Identity-by-state cluster analysis based on PLINK's hierarchical clustering algorithm was used to verify that the self-reported ethnicities were consistent with the clusters generated from the SNP marker data.

Consistent with the ethnicity distribution in our sampling area, the majority of our families, 403, were Caucasian, while there were only 26 African American families. For the purposes of analyses, the larger Caucasian data set was first analyzed by itself. Here, 147 SNPs were excluded due to a HWE p-value < = 0.001 (computed by PLINK using only founder genotypes); 1,936 SNPs were excluded because their genotyping success rate across all individuals was less than 90%; 999 SNPs were excluded due to an MAF < 0.05; leaving 8,802 SNPs that were used in the linkage analyses.

### Allele frequencies

For the initial analyses using Merlin [31], allele frequencies were estimated from the data, ignoring family structure, by simple gene counting. On small nuclear families such as we have here, this approach results in unbiased estimates [32]. For the subsequent analyses using Mendel [33], allele frequencies were estimated by maximizing the pedigree likelihood while properly taking the family structures into account.

### Linkage Analysis

We used PedCheck [34] to detect Mendelian inconsistencies; all genotypes at each problematic SNP were zeroed out within each family containing a Mendelian inconsistency. We used Merlin [31] to carry out non-parametric linkage analyses using the S_all _statistic using sex-averaged genetic maps and allele-counting allele frequency estimates; Minx was used to analyze the X-linked data. Summary statistics were computed using Pedstats [35]. File-format conversions were done using Mega2 [36].

To assay the effects of SNP-SNP linkage disequilibrium, we recomputed the LOD scores using Merlin's LD modeling (--rsq 0.1) option [37]. To examine robustness to other modeling assumptions, we also analyzed the data using the Mendel v.9.0.0 package [33] in a series of three steps: (a) We used Mendel's mistyping option to zero out all genotypes at each problematic SNP within each family containing a Mendelian inconsistency; (b) We used Mendel's allele frequency option to estimate the allele frequencies at each SNP while properly taking the family structures into account; (c) We used Mendel's non-parametric linkage (NPL) option to compute an 'additive all' linkage statistic, using the replicate pool method with 50 replicates and 100,000 samples to generate accurate empirical P-values. These analyses used sex-specific genetic maps for the autosomes, and the female map for the X chromosome.

### Combined linkage analyses

After the initial analyses using the Caucasian-only data set, we carried out additional linkage analyses of the combined Caucasian and African American data set. For these analyses, we used Mendel's options for estimating and using ethnic-group specific allele frequencies. We did not analyze the African American families by themselves, as the sample size was too small by itself to have adequate power: only 26 African American families containing 27 genotyped affected sib pairs.

### Candidate gene prediction

We used the GRAIL "Gene Relationships Across Implicated Loci" software to search our linkage peaks for possible candidate genes based on similarity to a set of previously implicated OM candidate genes [38]. Similarity is measured using automated statistical text mining of 250,000 PubMed abstracts. GRAIL can identify relatedness between genes even when there are no published co-citations or established pathways in common. GRAIL was run using a seed list of previously implicated candidate genes. While the GRAIL results do depend on the seed list, the GRAIL approach does avoid circular reasoning when assigning a score to each query region by not considering any seed regions that share genes with the query region.

### Association analyses

Using the Caucasian-only data set, we tested for association of 8,585 autosomal SNPs (which met our quality control criteria) using the gamete competition statistic implemented in Mendel [33]. This statistic, which tests the null hypothesis of no linkage and no association, uses all the family information to test for over-transmission of a specific SNP allele to affecteds with complementary under-transmission to unaffecteds. These results were annotated using the WGA Viewer program [39].

## Results

Using a stringent diagnostic model in which only those who underwent tympanostomy tube insertion at least once for recurrent/persistent OM are considered affected, we have carried out a genome-wide linkage scan using the 10K Affymetrix SNP panel. We genotyped a panel of 403 Caucasian families containing 1,431 genotyped individuals, and 26 African American families containing 75 genotyped individuals. For the purposes of analyses, the larger Caucasian data set was first analyzed by itself. After careful quality control, non-parametric linkage analysis was carried out using 8,802 SNPs; the cleaned Caucasian data contained 377 genotyped affected sib pairs (ASPs) (89 female-female ASPs, 110 male-male ASPs, and 178 male-female ASPs). Initial linkage analyses with Merlin found two regions with linkage peaks with linear S_all _LOD scores > 2.0 with a maximum LOD of (a) 2.83 on chromosome 17q12 at rs226088 and (b) 2.25 on 6p25.1 at rs554653 (Additional file 1). When marker-to-marker linkage disequilibrium (LD) is modeled, we obtain a maximum LOD of 2.85 at rs226088, and of 2.16 at rs554653. Additionally, we observed a maximum LOD of 2.00 on chromosome 7q33 at rs1343697 (increased from 1.96 previously). Sensitivity to marker-to-marker LD should be modest, as 57.6% of the families have both founders genotyped, and 38.5% have one founder genotyped.

**Figure 1:**
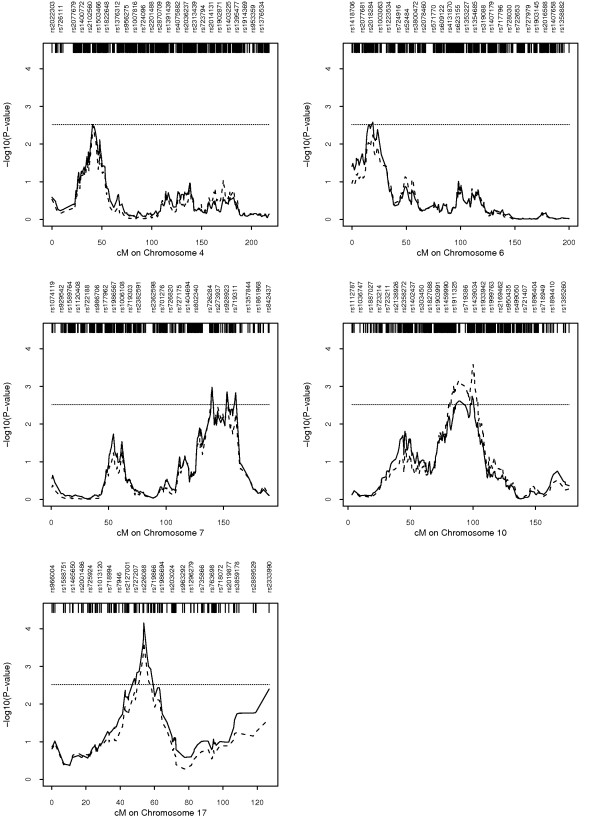
**The empirical -log10(p-values) for the Additive ALL statistics for the five highest peaks**. Solid line: Caucasian-only results; dashed line: combined Caucasian and African American results. The horizontal reference line indicates a nominal P-value of 0.003.

Mendel-based linkage results, which use better allele-frequency estimates, sex-specific maps, and empirical P-values, are summarized in Table 1 and Figure 1. For the Caucasian-only data set, the most significant empirical p-value on chromosome 17q12 is 0.00007 at rs226088. There are four other peaks with suggestive p-values: chromosome 10q22.3 with a minimal p-value of 0.00181 at rs1878001; chromosome 7q33 with a minimal p-value of 0.00105 at rs958408; chromosome 6p25.1 with a minimal empirical p-value of 0.00261 at rs554653; and chromosome 4p15.2 with a minimal p-value of 0.00301 at rs2133507.

**Table 1:** Additive ALL linkage statistics as computed by Mendel, grouped by peak, for the Caucasian-only data set and for the combined Caucasian and African American data set.

**Chromosome**	**Locus**	**Physical position**	**cM**	**Caucasian P-value**	**Combined P-value**
17	rs11439	30539277	53.18	**0.00028**	0.00062
17	rs938298	30711529	53.41	**0.00022**	**0.00047**
17	rs722374	30770914	53.48	**0.00020**	**0.00042**
17	rs226088	31041457	53.75	***0.00007***	***0.00023***
17	rs2680398	32663038	55.69	0.00052	0.00127
					
10	rs1878001	79048564	99.31	*0.00181*	**0.00039**
10	rs2244688	79076786	99.34	0.00183	**0.00039**
10	rs2812415	79076976	99.35	0.00185	**0.00039**
10	rs719871	79815441	100.11	0.00215	***0.00026***
10	rs1369752	79918761	100.23	0.00223	**0.00027**
					
7	rs1343697	132603523	139.97	0.00118	0.00115
7	rs718656	132771392	140.06	0.00106	*0.00110*
7	rs958408	132791458	140.08	*0.00105*	0.00111
7	rs1073158	133116548	140.27	0.00123	0.00128
7	rs958404	133247726	140.33	0.00127	0.00132
					
6	rs1112267	4873386	15.03	0.00330	0.00921
6	rs2326584	5234205	16.06	0.00304	0.00962
6	rs2326689	5966877	17.94	0.00312	0.00786
6	rs1902946	6313577	18.81	0.00276	0.00649
6	rs554653	6492486	19.23	*0.00261*	*0.00569*
					
4	rs2133507	24779140	40.67	*0.00301*	0.00521
4	rs1402031	25204628	41.72	0.00365	0.00469
4	rs939353	25204915	41.73	0.00366	0.00467
4	rs1378943	25382169	42.20	0.00339	*0.00461*
4	rs1378946	25382548	42.21	0.00338	0.00463

For each peak, we list the 5 SNPs that are most significant in the Caucasian data set. Bold indicates a p-value less than 0.0005; the minimum p-value in each region is in italics.

When we carried out analyses of the combined Caucasian and African American sample, using group-specific allele frequency estimates, three of the peaks (on chromosomes 17, 6, 4) became a bit less significant, while the chromosome 7 peak significance level stayed essentially the same and the chromosome 10 peak became more significant, with a minimal p-value of 0.00026 at rs719871 (Table 1 and Figure 1). As mentioned above, the combined sample adds 26 African American families containing 75 genotyped individuals. This African American sample contains 27 genotyped affected sib pairs (ASPs) (5 female-female ASPs, 8 male-male ASPs, and 14 male-female ASPs).

We used the GRAIL "Gene Relationships Across Implicated Loci" software to search our linkage peaks for possible candidate genes based on similarity to a 'seed' set of previously implicated OM candidate genes [38]. To do this, we used a seed list of 36 candidate genes (*ADAMTS13, BCL6, CD14, DNAH5, EVI1, EYA4, FBXO11, FCGR2A, GJB2, GJB6, HLA-A, IFNG, IL10, IL13, IL1A, IL1RN, IL4R, IL6, MAPK14, MAPK8, MBL2, MMP2, MMP9, MUC2, MUC4, MUC5AC, MUC5B, SERPINE1, SERPING1, SFTPA1B, SFTPA2B, SFTPD, TGFB1, TLR2, TLR4, TNF*). For the chromosome 17 linkage peak, the most significant candidate genes with p-values < 10^-13 ^are *CCL5*, *CCL18*, *CCL3*, and *CCL4*. For the chromosome 10 peak, the most significant candidate genes with p-values < 10^-13 ^are *SFTPA1B*, *SFTPA2*, *SFTPD*, and *PLAU*. For the chromosome 7 peak, the most significant candidate gene *EPHB6 *had a p-value of 2.6 × 10^-6^. For the chromosome 6 peak, the most significant candidate genes with p-values < 10^-11 ^are *LY86*, *SERPINB9*, and *F13A1*. For the chromosome 4 peak, the most significant candidate gene with p-values < 10^-11 ^was *SOD3*.

Using the Caucasian-only data set, we tested for association of 8,585 autosomal SNPs (which met our quality control criteria) using the gamete competition statistic implemented in Mendel [33]. The most significant association signals are listed in Additional file 2. While none of these are significant after Bonferroni or false discovery rate correction for multiple testing, there are a few highly ranked signals that are near previously implicated candidate genes. One SNP (rs1437803, P-value 0.0005, rank 5) is 513 kb from *SFTPA2 *on chromosome 10. Two SNPs (rs722749, P-value 0.0009, rank 11 and rs722748, P-value 0.0013, rank 15) are 48 kb from *IFNG *on chromosome 12. Two SNPs (rs2213584, P-value 0.0021, rank 22 and rs2227139, P-value 0.0021, rank 23) are 870 kb from *TNF *on chromosome 6. Note that the gamete competition test should detect association signals at much larger distances than a regular case/control association test in unrelateds because the gamete competition test relies on measuring transmission distortion within families.

## Discussion

While there is fairly strong evidence that otitis media has a strong genetic component, to our knowledge there has been only one prior family-based linkage study of otitis media. This first study provided evidence of linkage of COME/ROM to chromosome 10q26.3 at marker D10S212 and to chromosome 19q13.43 at marker D19S254 [20]. We report here on our genome-wide linkage scan for risk genes for otitis media. In our study, we chose to focus on severe disease, collecting affected sib pair families where at least two full siblings had undergone tympanostomy tube insertion. By narrowing the disease definition, we hoped to identify a more severe form of OM that is perhaps due to a small number of loci. This approach has proven remarkably successful in several complex diseases, such as breast cancer and Alzheimer's disease, where subdivision according to age of onset contributed to the mapping and cloning disease genes [40,41]. Additionally, it has been shown that there are statistical advantages in using a "narrow-phenotype" approach [42], because the power of an affected sib pair approach increases as the population prevalence of the trait decreases.

The rate of tympanostomy tube insertion in second siblings may be increased due to the parents' wishes to have the procedure performed earlier or for less significant disease than the first child because of prior positive experiences in the first sibling. This could potentially make it difficult to separate the genetic effect of OM from the parents' influence on the treatment. However, we have reason to believe such an effect, if it exists, will be small in our sample. Firstly, the majority of our sample was treated at the Children's Hospital of Pittsburgh, where our criteria for insertion for tubes are consistent and very stringent. Secondly, at entry we obtained, for each subject, a history of middle-ear disease prior to tube insertion to assess the subject's eligibility.

Since the majority of our families were Caucasian, we present two linkage analyses, one where we analyzed only the 403 Caucasian families by themselves, and a combined analysis where we jointly analyzed both the 403 Caucasian and 26 African American families (Table 1 and Figure 1). The Caucasian-only analyses generate a strong linkage signal on chromosome 17q12, as well as four other potentially interesting peaks (10q22.3, 7q33, 6p25.1, 4p15.2). The combined analyses strengthen the evidence for the 10q22.3 peak. Our scan does not provide evidence for linkage in the previously reported regions of 10q26.3 and 19q13.43 [20,21].

The very tip of our 17q12 linkage peak occurs in *AP2B1 *(adaptor-related protein complex 2, beta 1 subunit), which plays a role in Nef-mediated CD8 down-regulation [43], and children with recurrent otitis media had low numbers of CD8+-producing IFN g cells in adenoids [44]. However, the chromosome 17 peak also contains a cluster of *CCL *(chemokine C-C motif ligand) genes, several of which were highlighted as possible candidates by the GRAIL analyses. *CCL5*, also known as *RANTES*, is 18 kb from the linkage peak, and has been previously associated with otitis media [45-52]. *CCL5 *is an eosinophil chemoattactrant that is thought to play a role in the accumulation of eosinophils often observed in middle ear effusions of OM with allergy.

The combined 10q22.3 linkage peak is 1.2 Mb from a previously implicated candidate gene *SFTPA2*. This peak also contains a strongly associated SNP rs1437803 (Additional file 2, P-value 0.0005, rank 5), which is 513 kb from *SFTPA2*. The human surfactant protein A (SP-A) is expressed in the Eustachian tube, plays a role in innate host defense, upregulates phagocytosis of many OM risk pathogens (including *Streptococus pneumoniae, Haemophilus influenzae*, and respiratory syncytial virus), and consists of two very similar functional genes *SFTPA1 *and *SFTPA2 *located 5 kb apart on chromosome 10. Ramet et al [53] reported that the frequency of specific SP-A haplotypes and genotypes differ between children who experience their first episode of acute otitis before age 6 months and the general population. Pettigrew et al [54] also found that polymorphisms within the SP-A loci were protective for otitis media among white infants at risk for asthma.

In the remaining linkage peaks, the majority of the genes highlighted by GRAIL as possible candidates do not have prior evidence of involvement with OM.

While none of the association results (Additional file 2) are significant after correction for multiple testing, the level of evidence required is less for previously implicated genes. Thus, in addition to the associated SNP 513 kb from *SFTPA2 *mentioned above, it is noteworthy that we have associated SNPs 48 kb from interferon-γ (IFNG) on chromosome 12. Genetic variants at *IFNG *are associated with risk for otitis media in infants infected with the respiratory syncytial virus (RSV) [55]. When RSV-induced and non-RSV-induced otitis media cases were compared, significantly higher levels of IFNG were found in the RSV-induced cases [56]. We also observed two associated SNPs 870 kb from tumor necrosis factor (*TNF*) on chromosome 6. A *TNF *-308 polymorphism was associated with both otitis media susceptibility and placement of tympanostomy tubes [57]. Variants in the promoter region of *TNF *were associated with being otitis-prone [58].

The gamete competition test of association used here relies on transmission distortion within families, and so should detect association signals at larger distances than the conventional case/control association test in unrelateds. Even so, the proximity of some association signals to previously implicated candidate genes mentioned above may not be that meaningful, as these regions contain many other plausible candidate genes.

## Conclusion

Our linkage scan, the largest to date, has identified two strong linkage peaks, on 17q12 and 10q22.3 that are likely to contain genes which influence risk for severe OM. While both of these peaks contain intriguing and plausible candidate genes, further fine-mapping, replication, and functional studies are required to reach more firm conclusions. We also find association signals near previously implicated risk genes (*SFTPA2*, *IFNG*, and *TNF*). Our hope is that our genetic results will help contribute to an enhanced understanding of the etiology of otitis media that ultimately may help lead to improved treatment and prevention of this extremely common childhood disease.

## Availability

The URLs for data presented herein are as follows:

PREST, 

RELPAIR, 

PLINK, 

Ensembl, 

Rutgers Combined Linkage-Physical Map, 

PedCheck, 

Pedstats, 

Merlin, 

Mega2, 

Mendel, 

Online Mendelian Inheritance in Man (OMIM), 

WGA Viewer, 

GRAIL, 

## Competing interests

The authors declare that they have no competing interests.

## Authors' contributions

MLC, EMM, REF, and DEW conceived of the study and obtained funding; KT, EMM, and MLC carried out the clinical work; REF supervised the genotyping; JPS, JJ, AR, and DEW carried out the statistical analyses; DEW, MLC, REF, and EMM wrote the initial draft of the manuscript; all authors read and approved the manuscript.

## Authors' information

Current affiliations: JJ: Department of Medical and Molecular Genetics, Center for Computational Biology and Bioinformatics, Indiana University, School of Medicine, Indianapolis, IN, 46202, USA; JPS: Eating Disorders Program, Department of Psychiatry, School of Medicine, University of North Carolina at Chapel Hill, Chapel Hill, NC, 27599, USA; AR: Statistics, reporting, and analysis (Cardiac Rhythm Management division), St. Jude Medical, Sunnyvale, CA, 94086, USA.

## Pre-publication history

The pre-publication history for this paper can be accessed here:



## Supplementary Material

Additional file 1 **Supplemental Figure S1**. This file provides multipoint linkage curves as computed by Merlin with and without linkage disequilibrium. Click here for file

Additional file 2 **Supplemental Table S1**. This file provides the gamete competition results in the Caucasian families for those SNPs with p-values < 0.01. Click here for file
