# Supplementary material for: Otitis media: a genome-wide linkage scan with evidence of susceptibility loci within the 17q12 and 10q22.3 regions
Source: BMC Med Genet. 2009 Sep 3;10:85. doi: 10.1186/1471-2350-10-85 (PMC2751750; doi:10.1186/1471-2350-10-85)

Supplemental Figure S1

Otitis media: a genome-wide linkage scan with evidence of susceptibility loci within the 17q12 and 10q22.3 regions

Margaretha L. Casselbrant, Ellen M. Mandel, Jeesun Jung, Robert E. Ferrell, Kathy Tekely, Jin P. Szatkiewicz, Amrita Ray, Daniel E. Weeks

Supplemental Figure S1: S_{All} LOD scores versus cM position on each chromosome, as computed using Merlin with and without linkage disequilibrium (LD) modeling.

Casselbrant et al (2009) Otitis media: a genome-wide linkage scan with evidence of susceptibility loci within the 17q12 and 10q22.3 regions

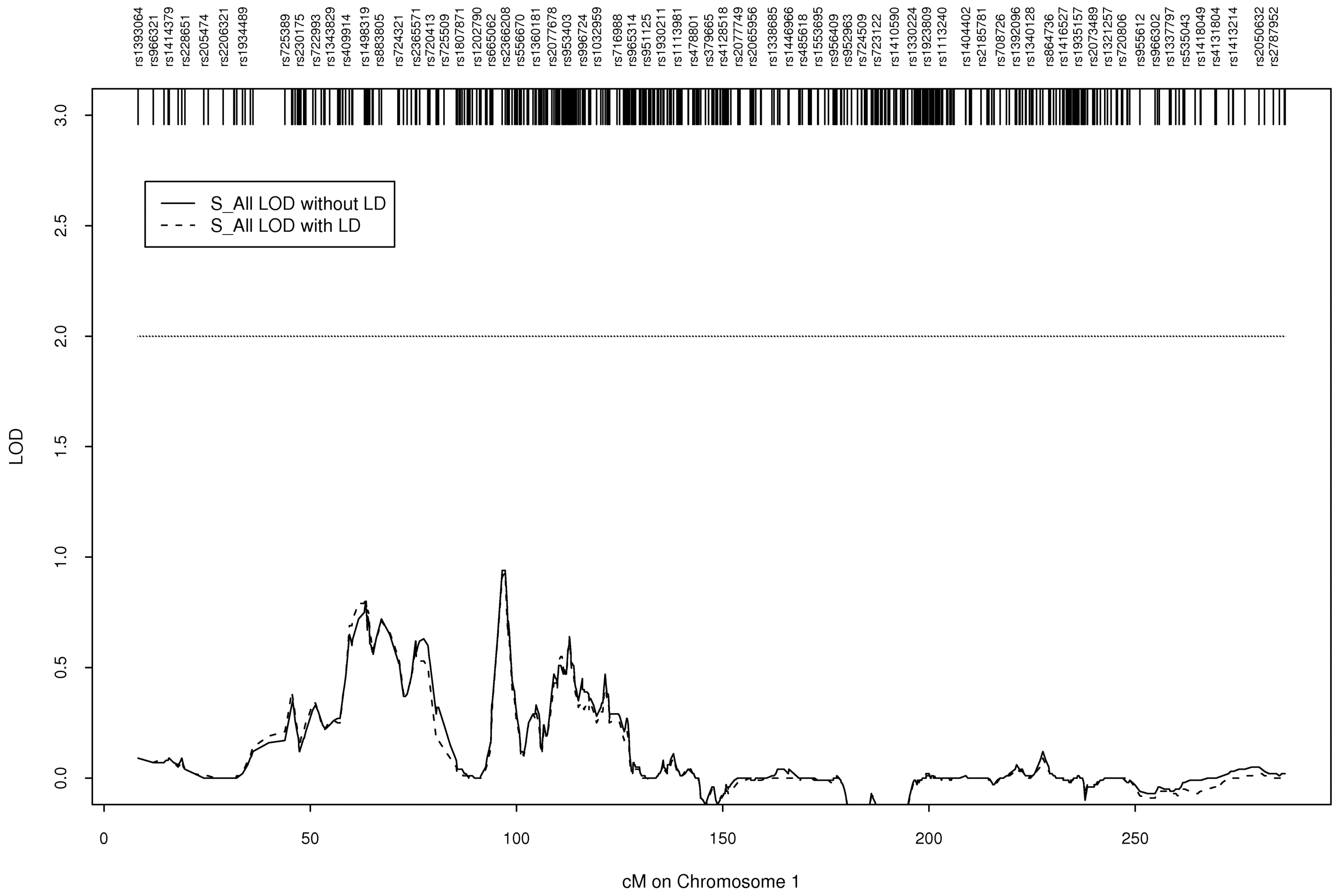

Casselbrant et al (2009) Otitis media: a genome-wide linkage scan with evidence of susceptibility loci within the 17q12 and 10q22.3 regions

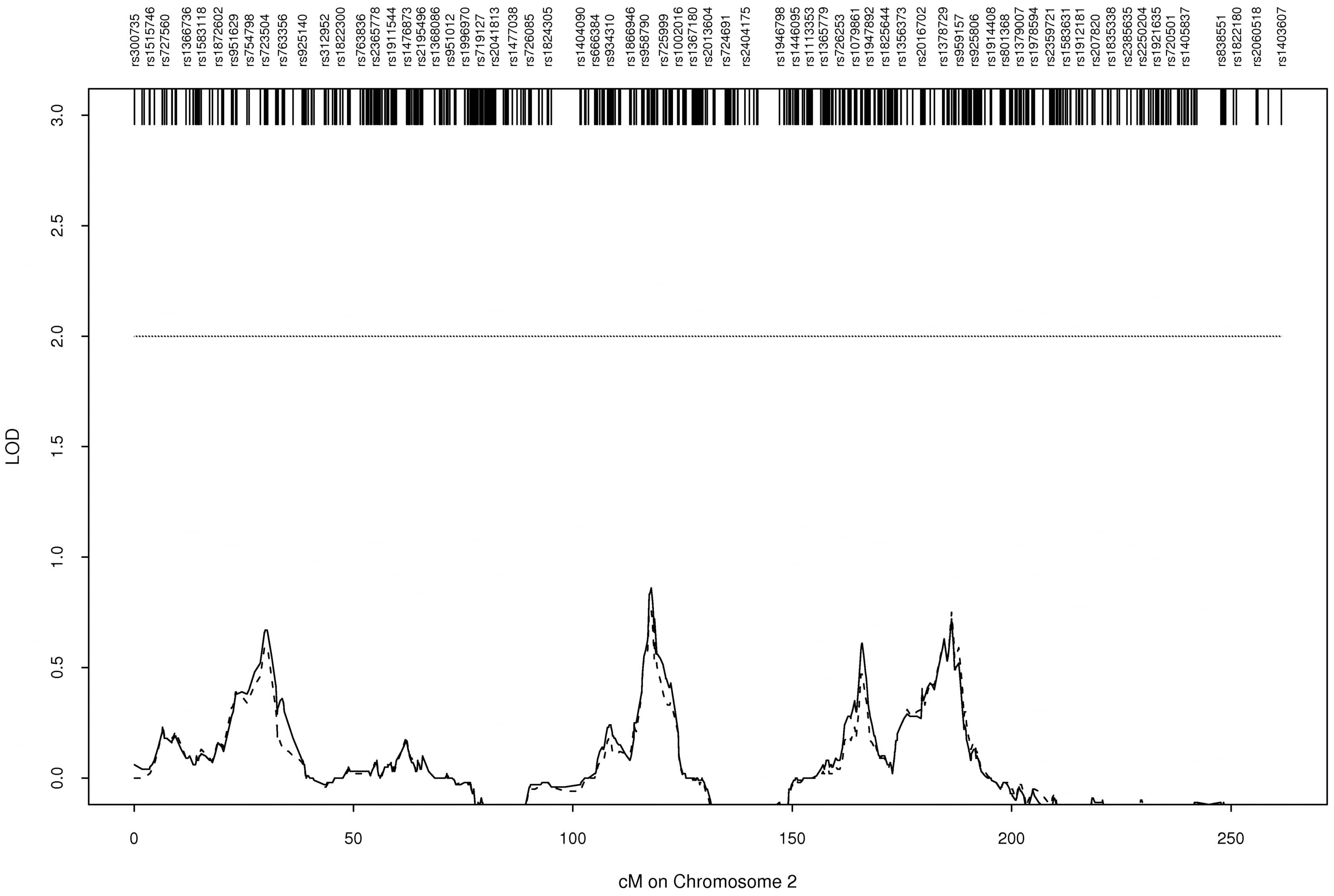

Casselbrant et al (2009) Otitis media: a genome-wide linkage scan with evidence of susceptibility loci within the 17q12 and 10q22.3 regions

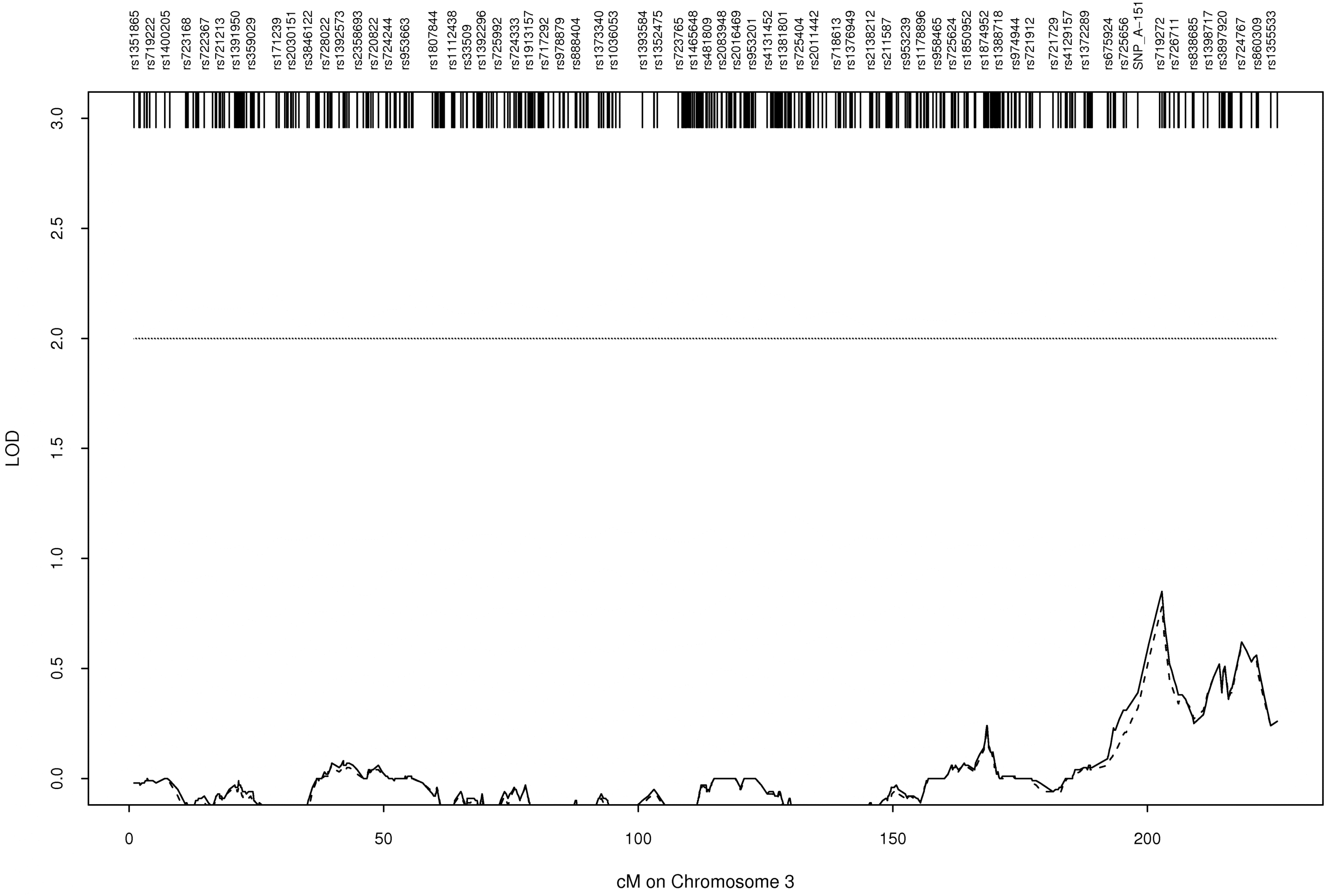

Casselbrant et al (2009) Otitis media: a genome-wide linkage scan with evidence of susceptibility loci within the 17q12 and 10q22.3 regions

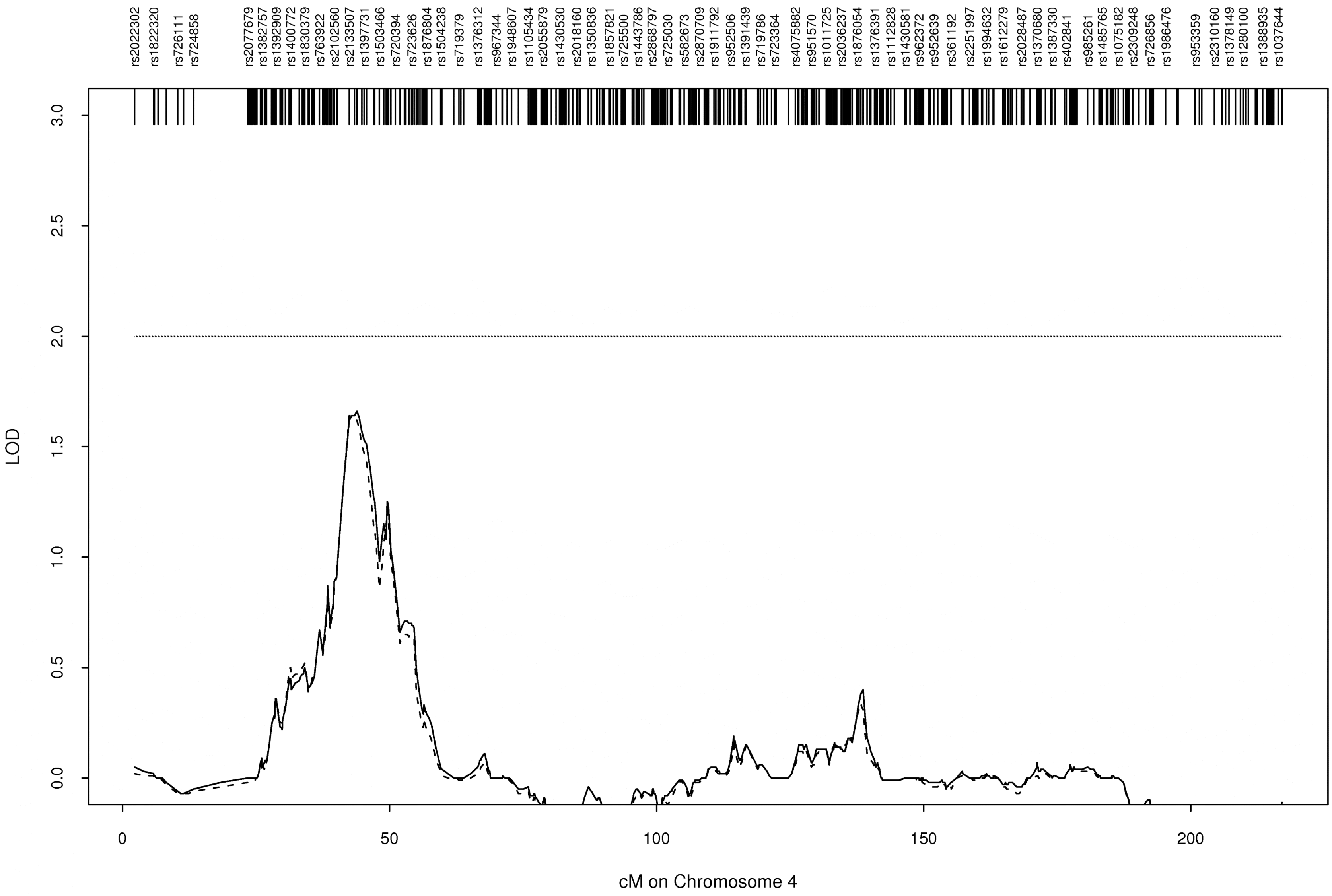

Casselbrant et al (2009) Otitis media: a genome-wide linkage scan with evidence of susceptibility loci within the 17q12 and 10q22.3 regions

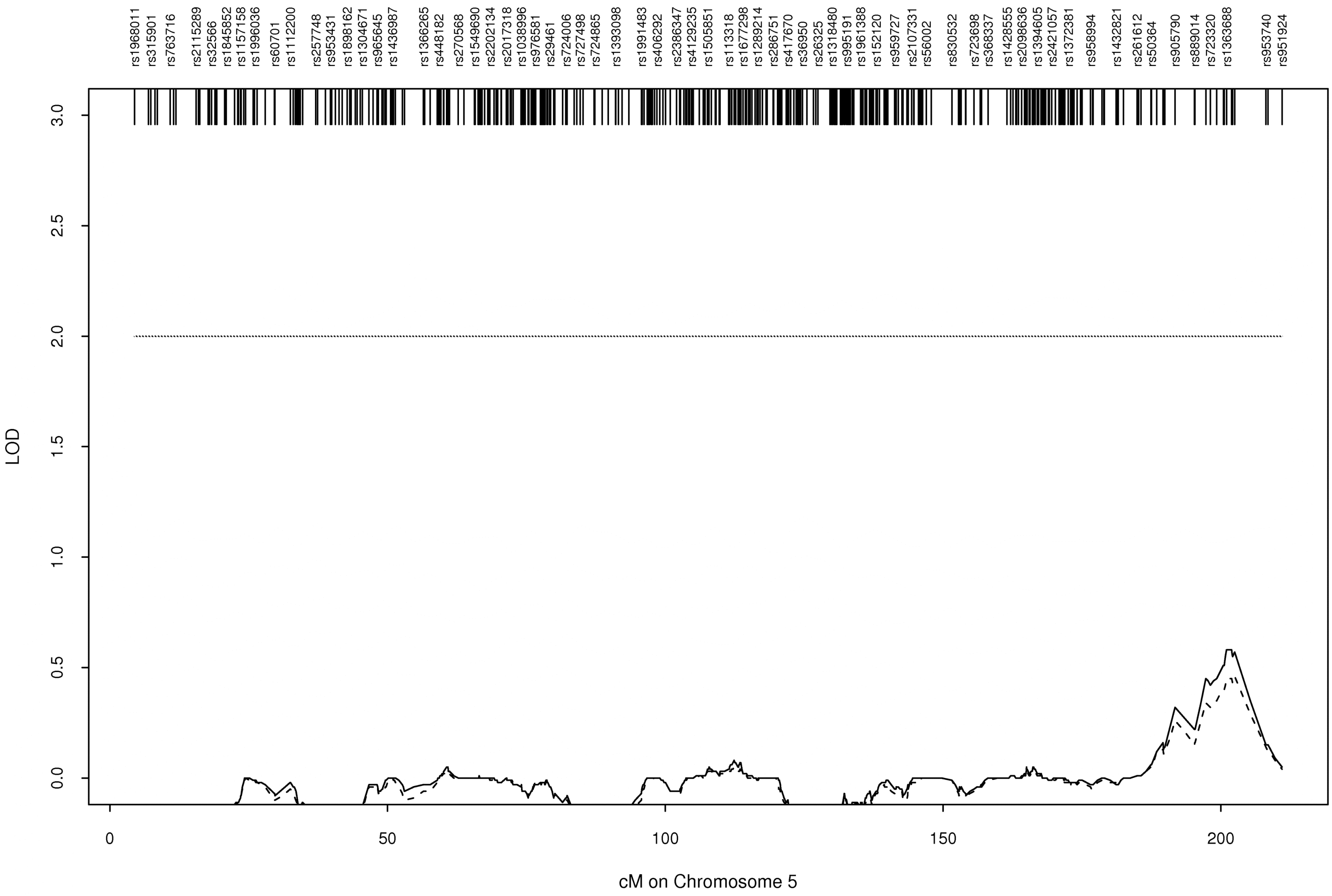

Casselbrant et al (2009) Otitis media: a genome-wide linkage scan with evidence of susceptibility loci within the 17q12 and 10q22.3 regions

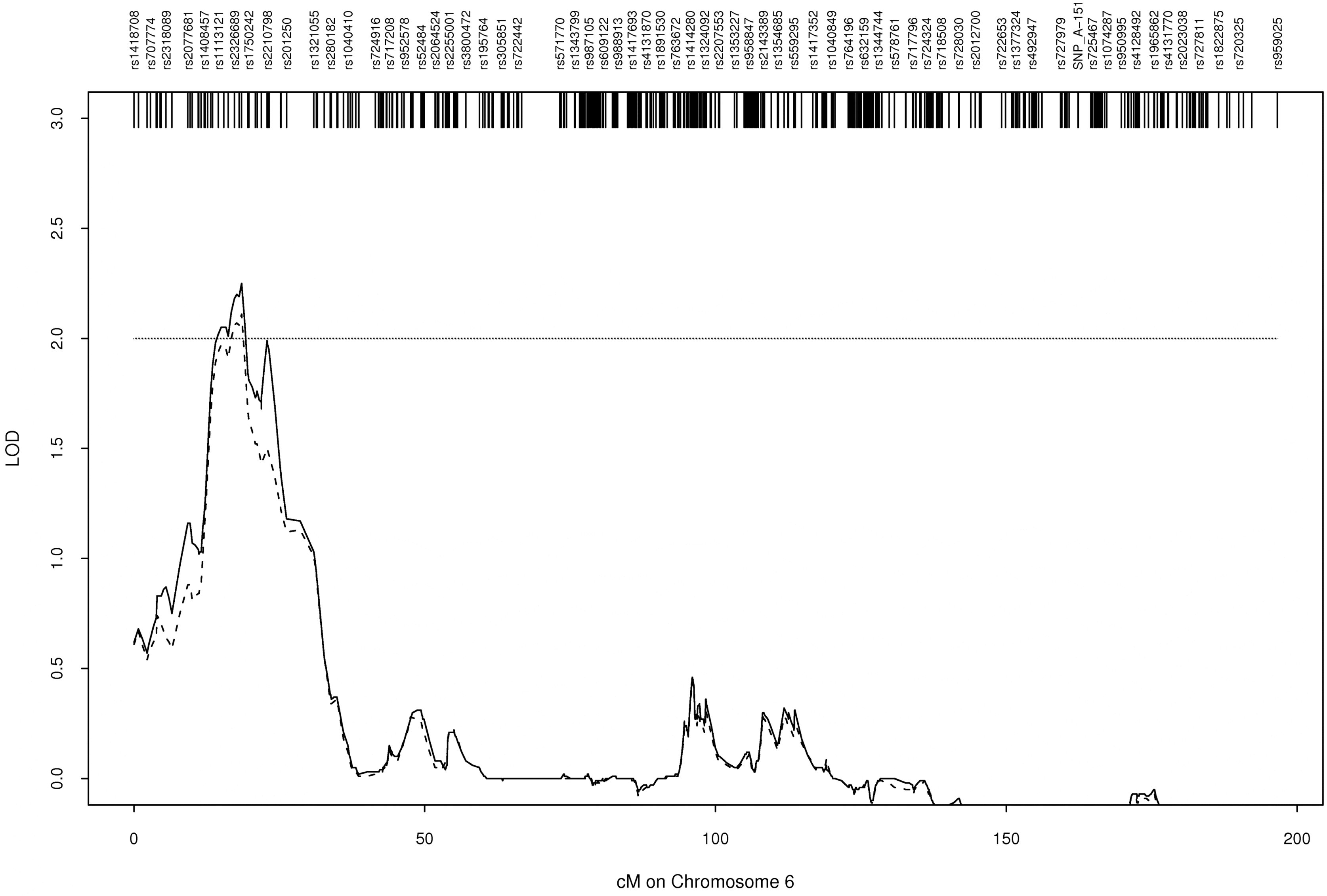

Casselbrant et al (2009) Otitis media: a genome-wide linkage scan with evidence of susceptibility loci within the 17q12 and 10q22.3 regions

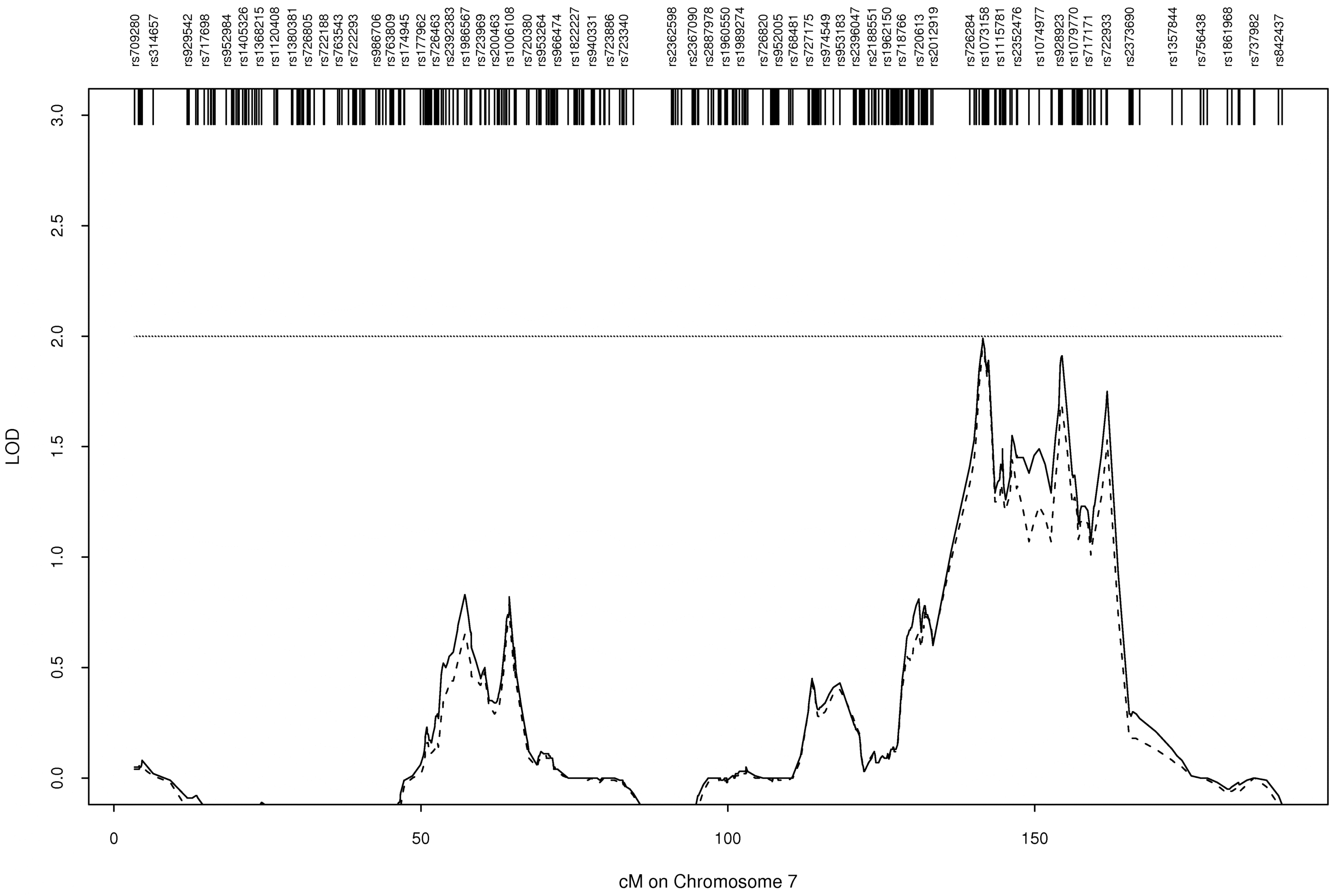

Casselbrant et al (2009) Otitis media: a genome-wide linkage scan with evidence of susceptibility loci within the 17q12 and 10q22.3 regions

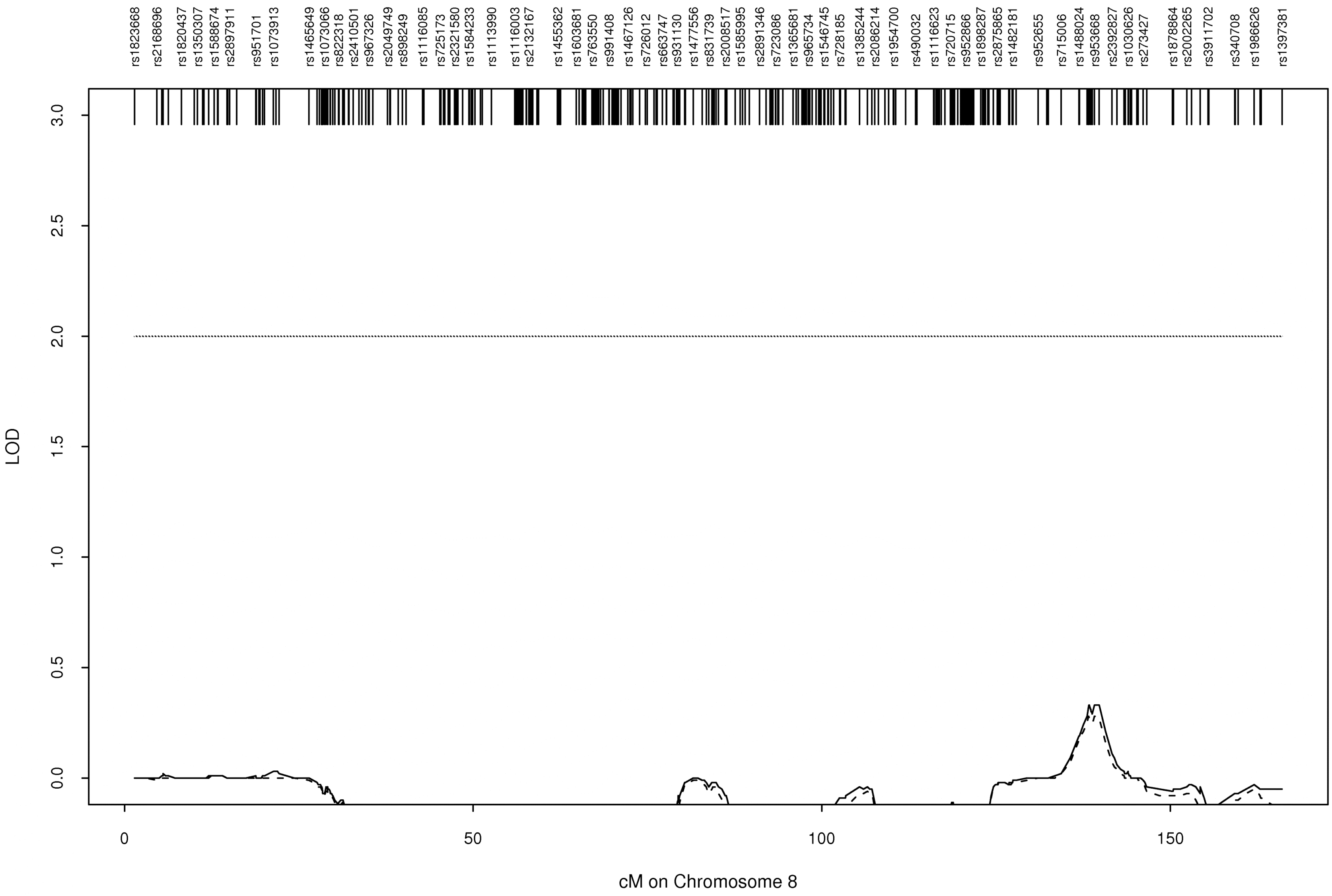

Casselbrant et al (2009) Otitis media: a genome-wide linkage scan with evidence of susceptibility loci within the 17q12 and 10q22.3 regions

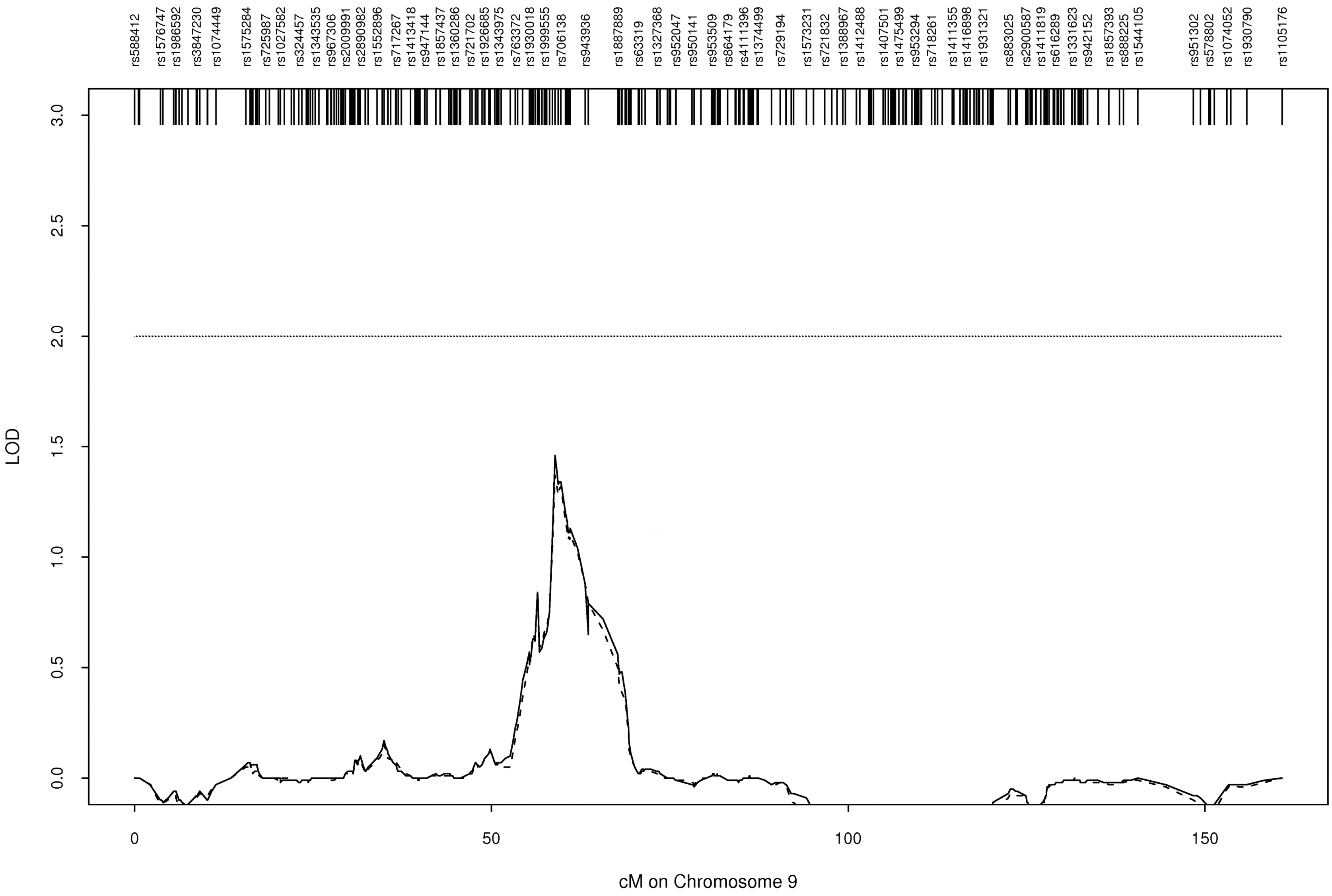

Casselbrant et al (2009) Otitis media: a genome-wide linkage scan with evidence of susceptibility loci within the 17q12 and 10q22.3 regions

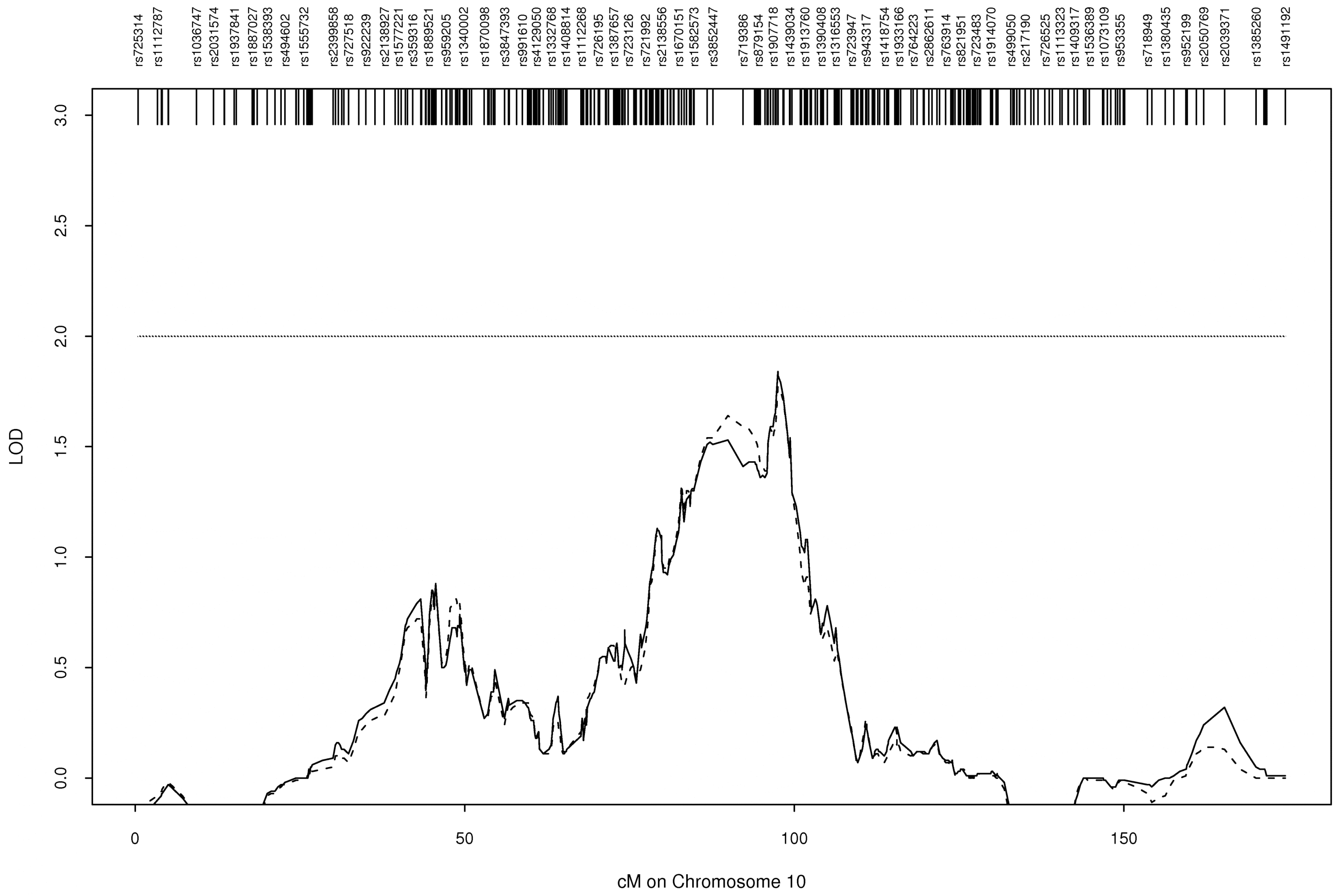

Casselbrant et al (2009) Otitis media: a genome-wide linkage scan with evidence of susceptibility loci within the 17q12 and 10q22.3 regions

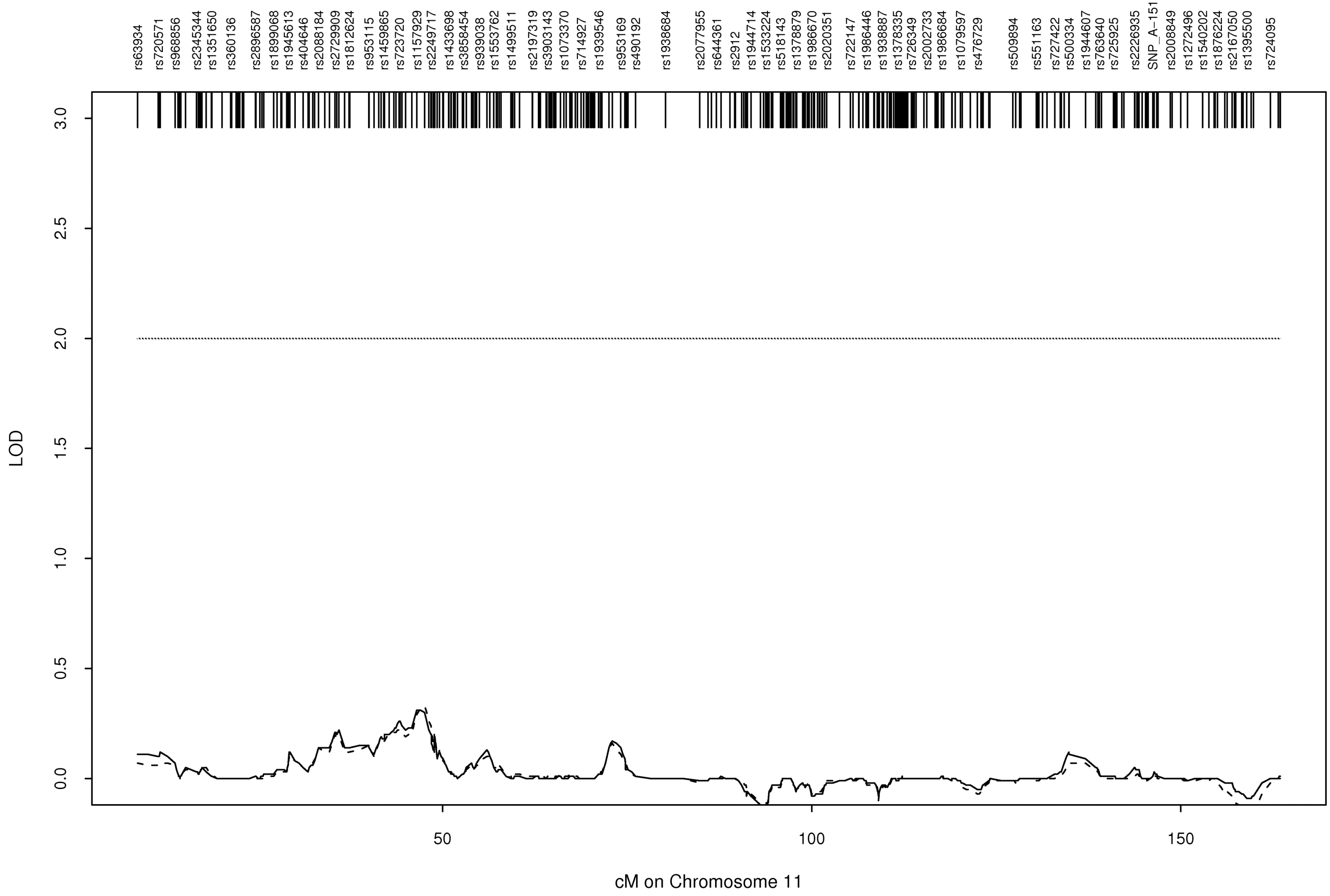

Casselbrant et al (2009) Otitis media: a genome-wide linkage scan with evidence of susceptibility loci within the 17q12 and 10q22.3 regions

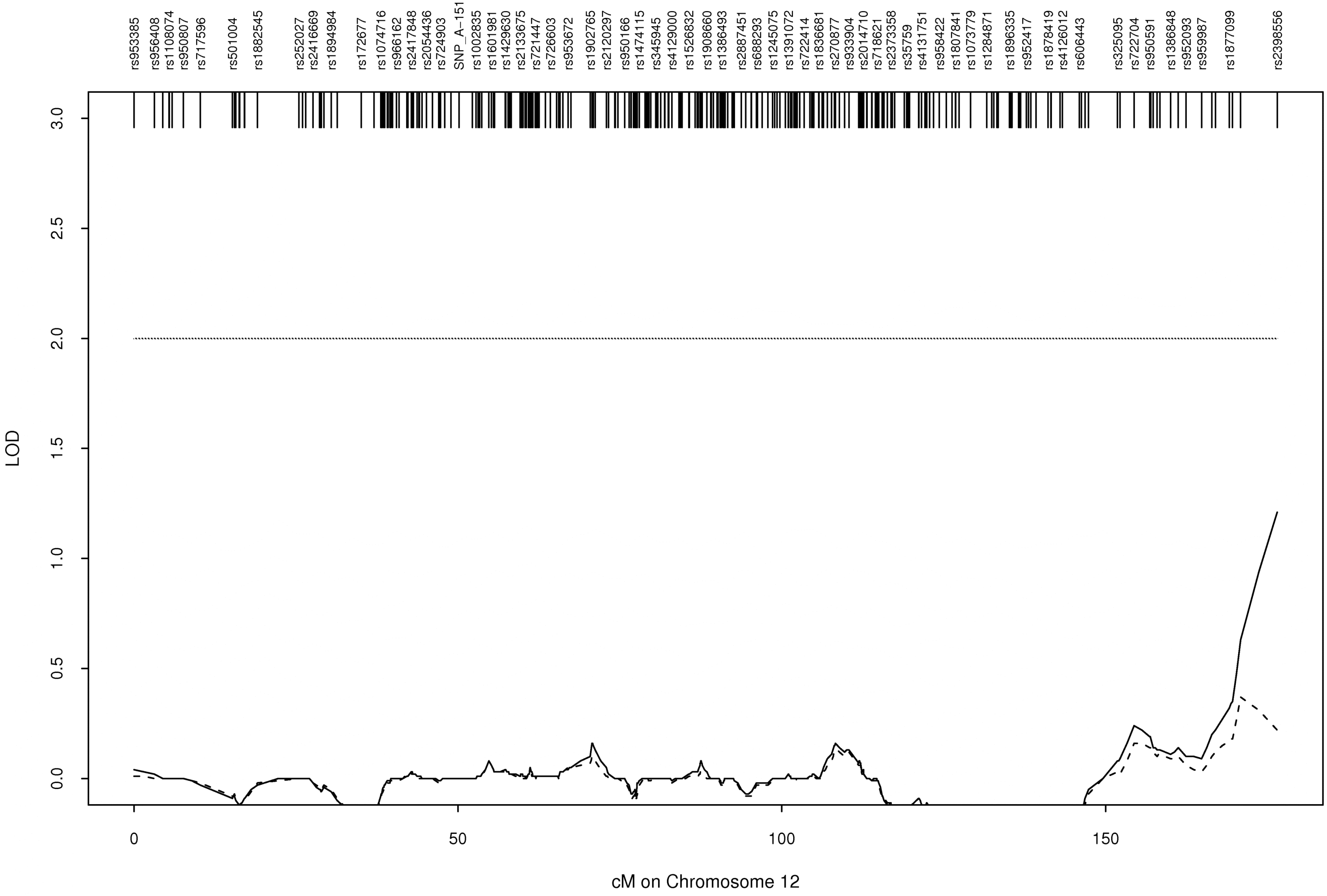

Casselbrant et al (2009) Otitis media: a genome-wide linkage scan with evidence of susceptibility loci within the 17q12 and 10q22.3 regions

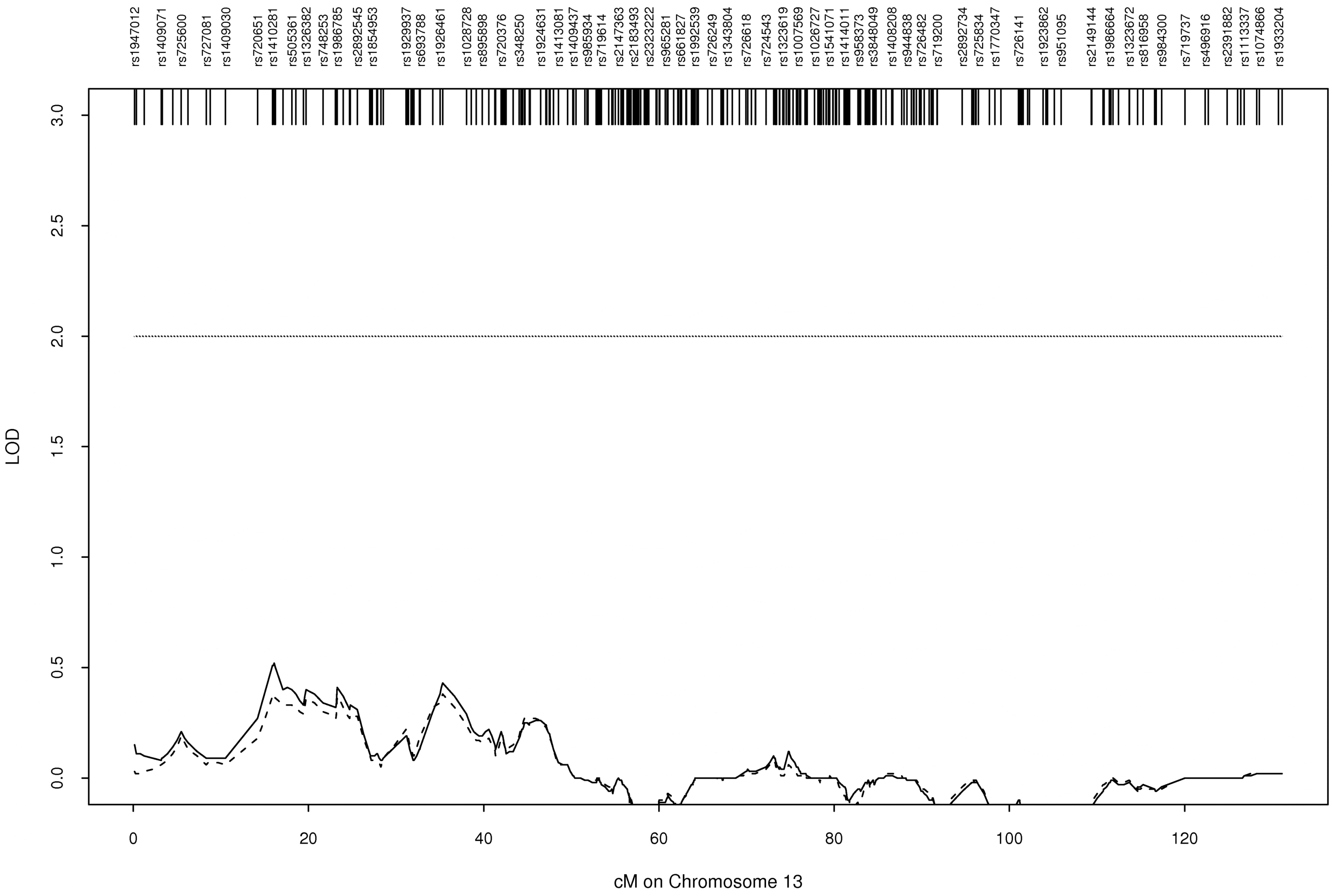

Casselbrant et al (2009) Otitis media: a genome-wide linkage scan with evidence of susceptibility loci within the 17q12 and 10q22.3 regions

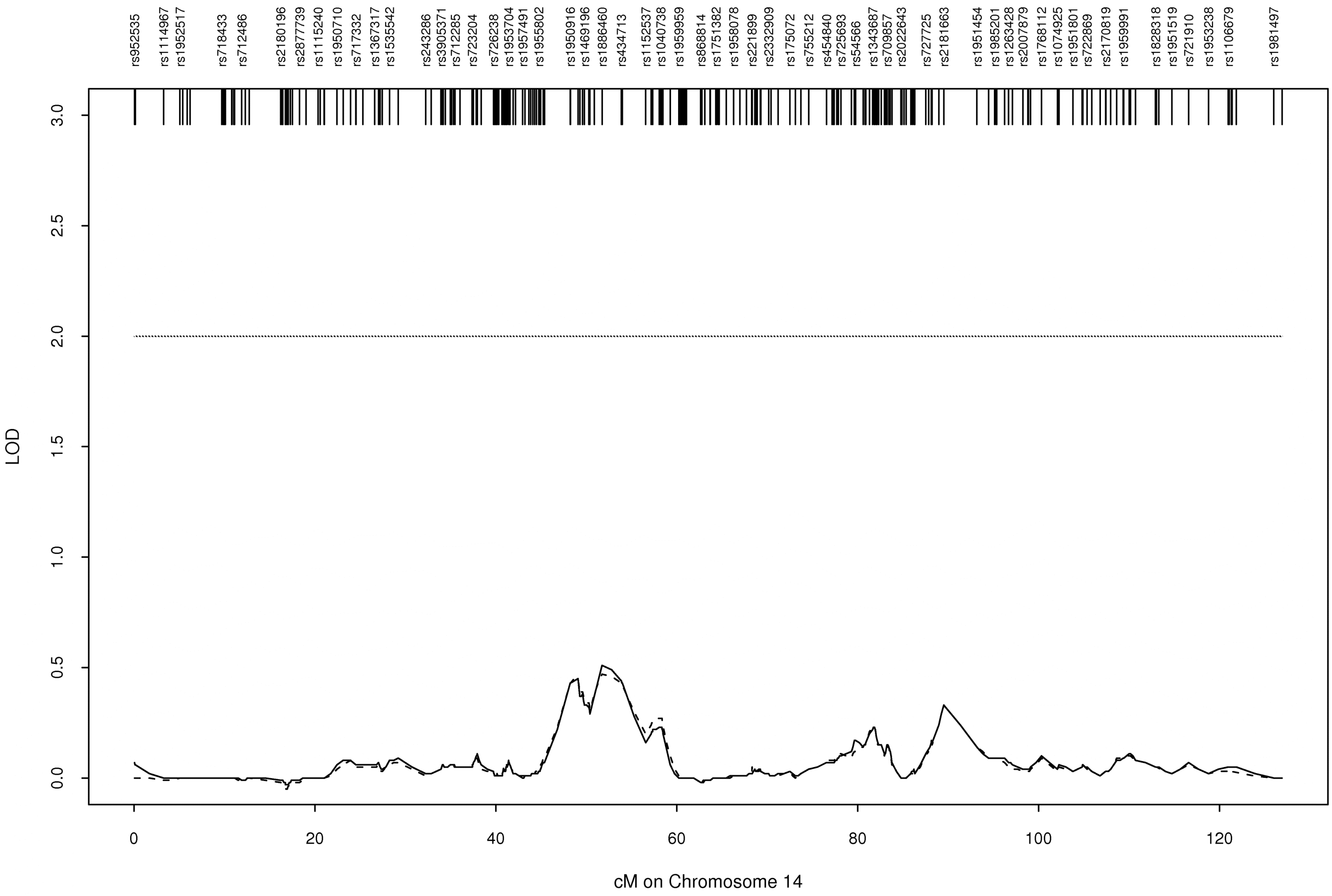

Casselbrant et al (2009) Otitis media: a genome-wide linkage scan with evidence of susceptibility loci within the 17q12 and 10q22.3 regions

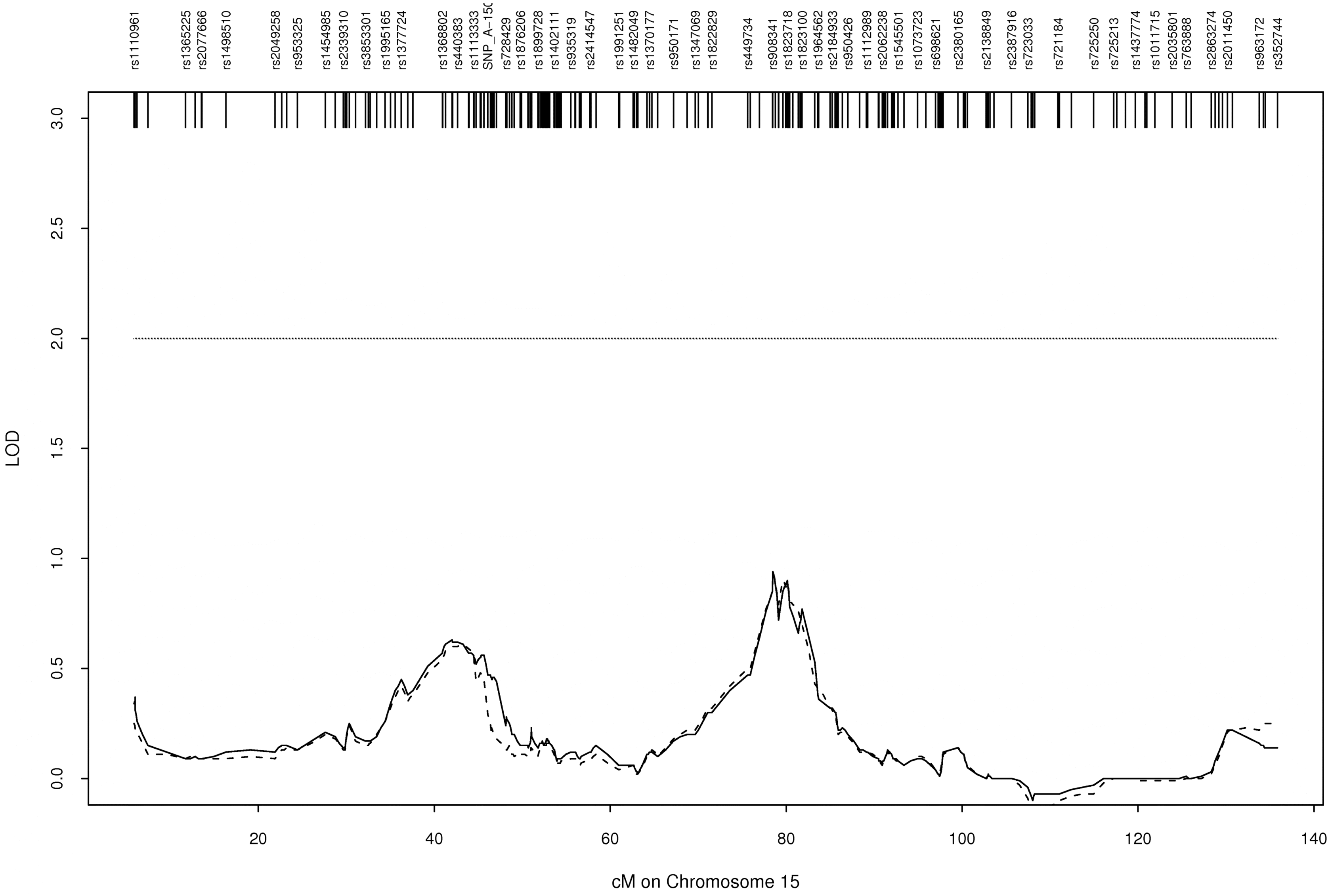

Casselbrant et al (2009) Otitis media: a genome-wide linkage scan with evidence of susceptibility loci within the 17q12 and 10q22.3 regions

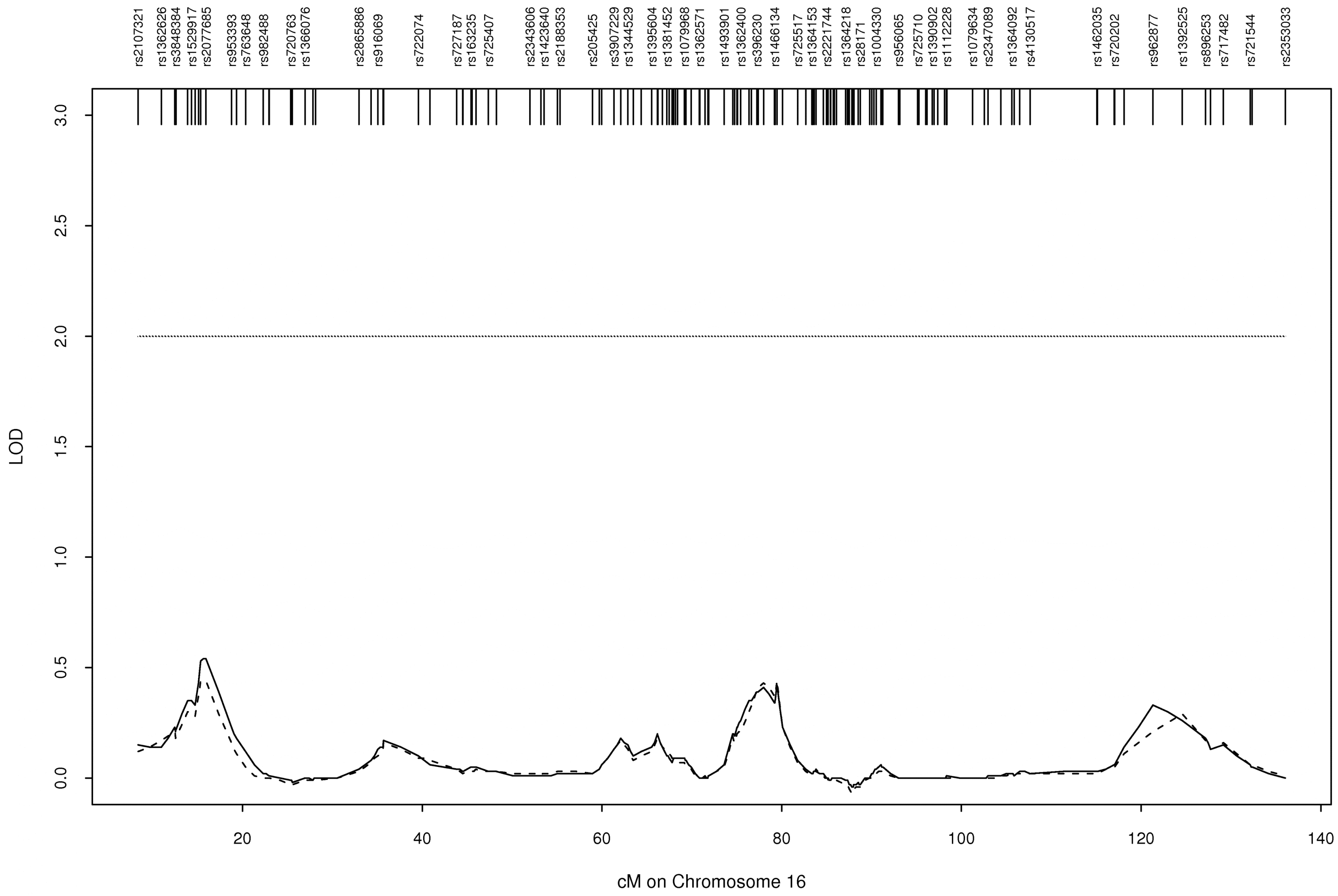

Casselbrant et al (2009) Otitis media: a genome-wide linkage scan with evidence of susceptibility loci within the 17q12 and 10q22.3 regions

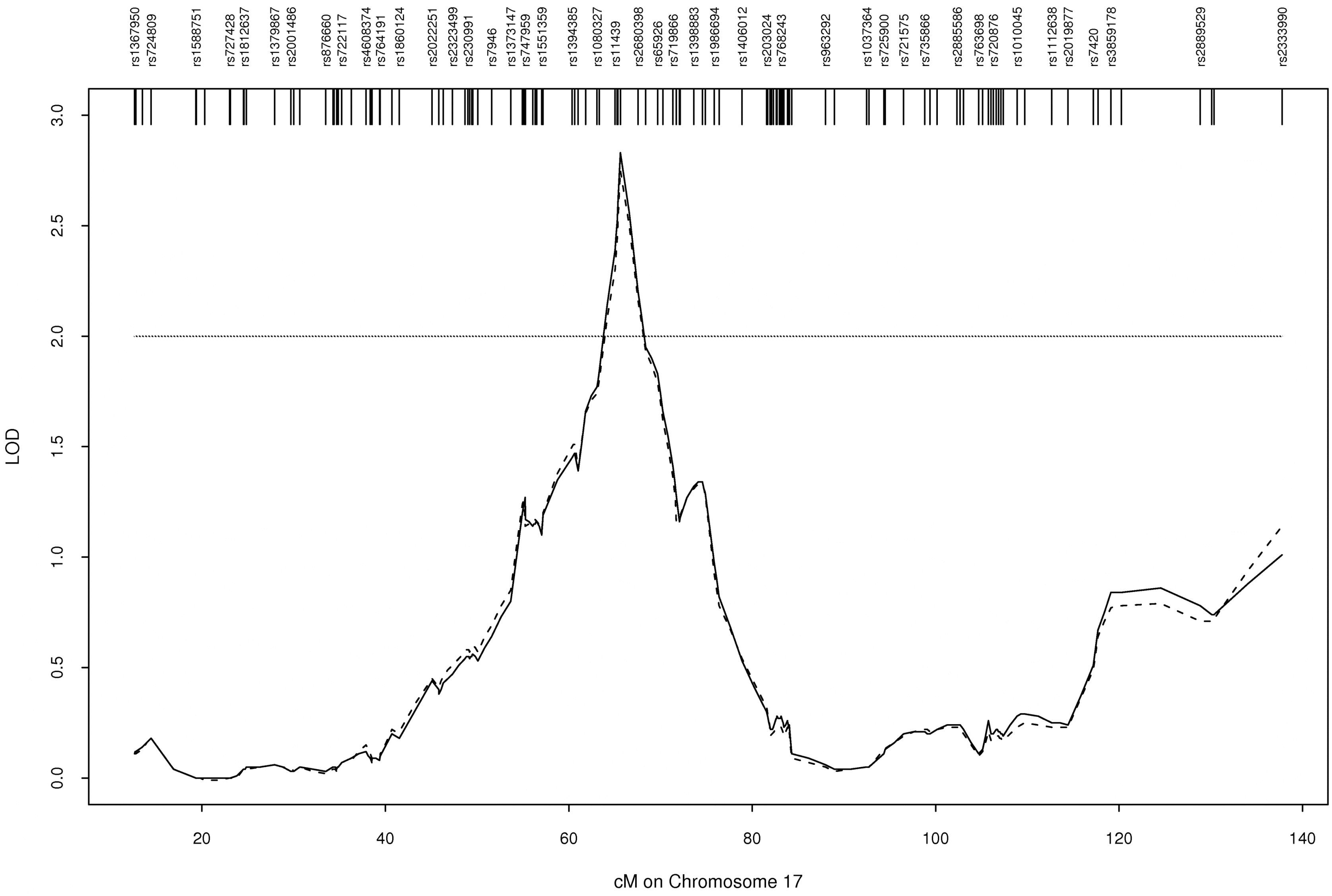

Casselbrant et al (2009) Otitis media: a genome-wide linkage scan with evidence of susceptibility loci within the 17q12 and 10q22.3 regions

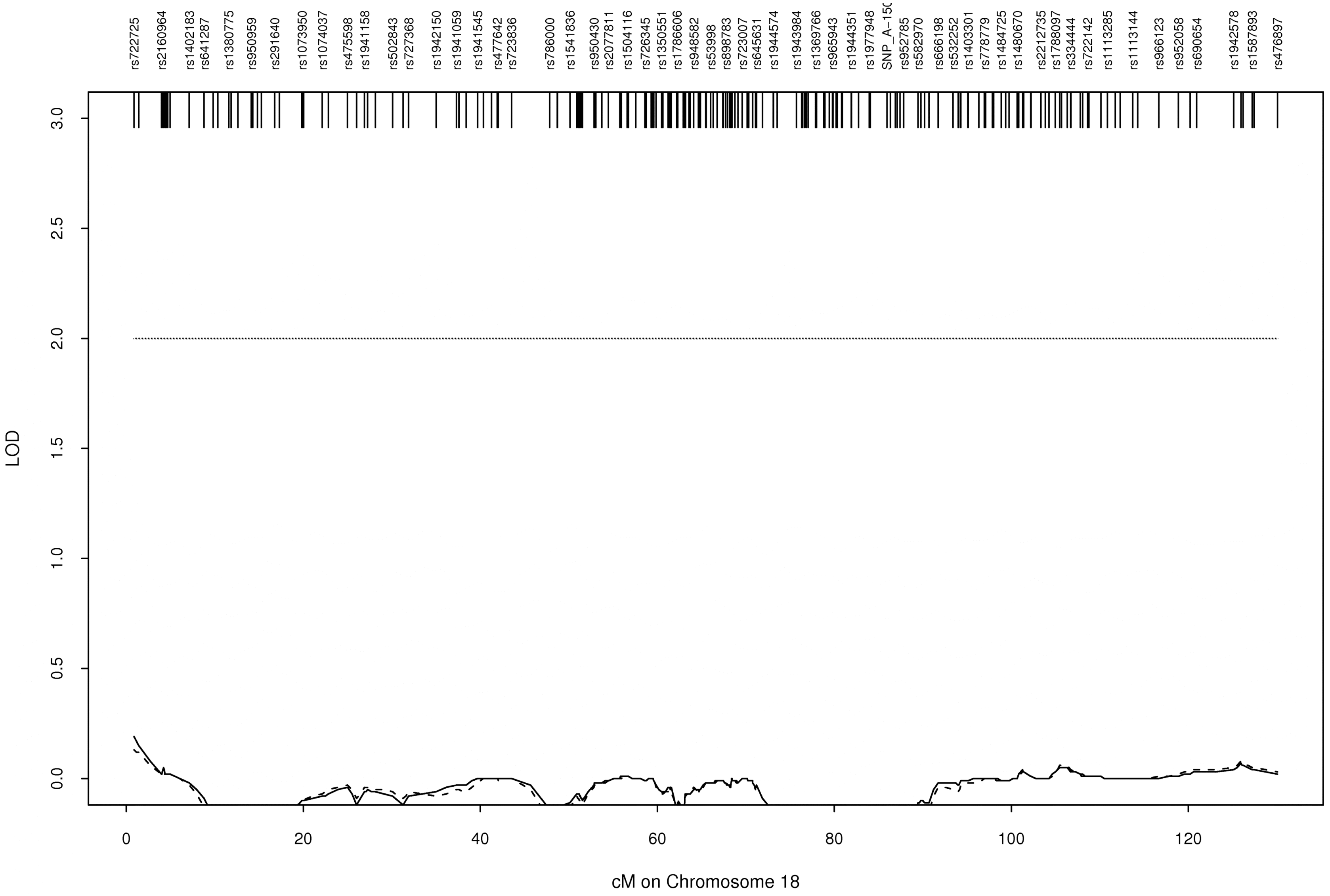

Casselbrant et al (2009) Otitis media: a genome-wide linkage scan with evidence of susceptibility loci within the 17q12 and 10q22.3 regions

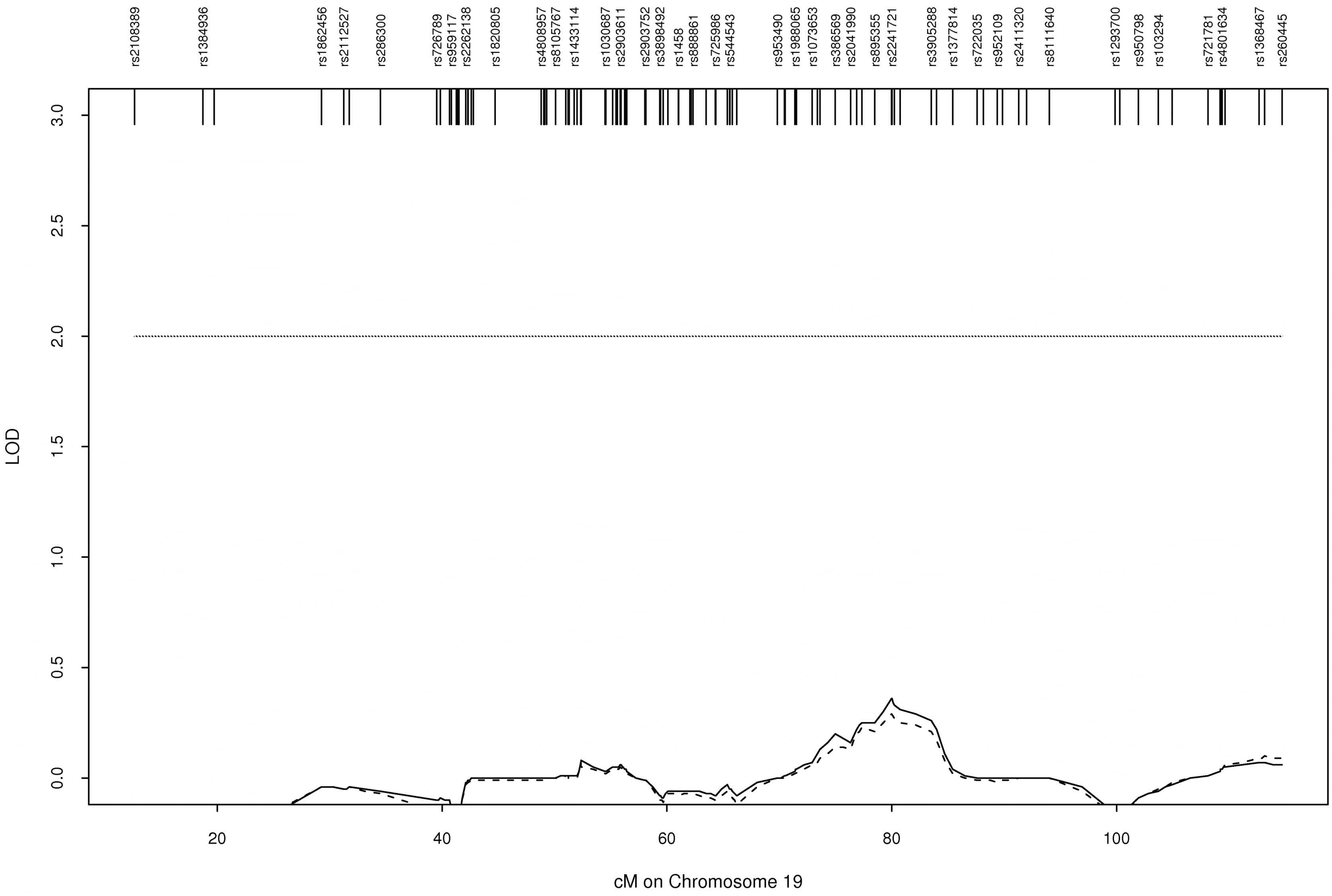

Casselbrant et al (2009) Otitis media: a genome-wide linkage scan with evidence of susceptibility loci within the 17q12 and 10q22.3 regions

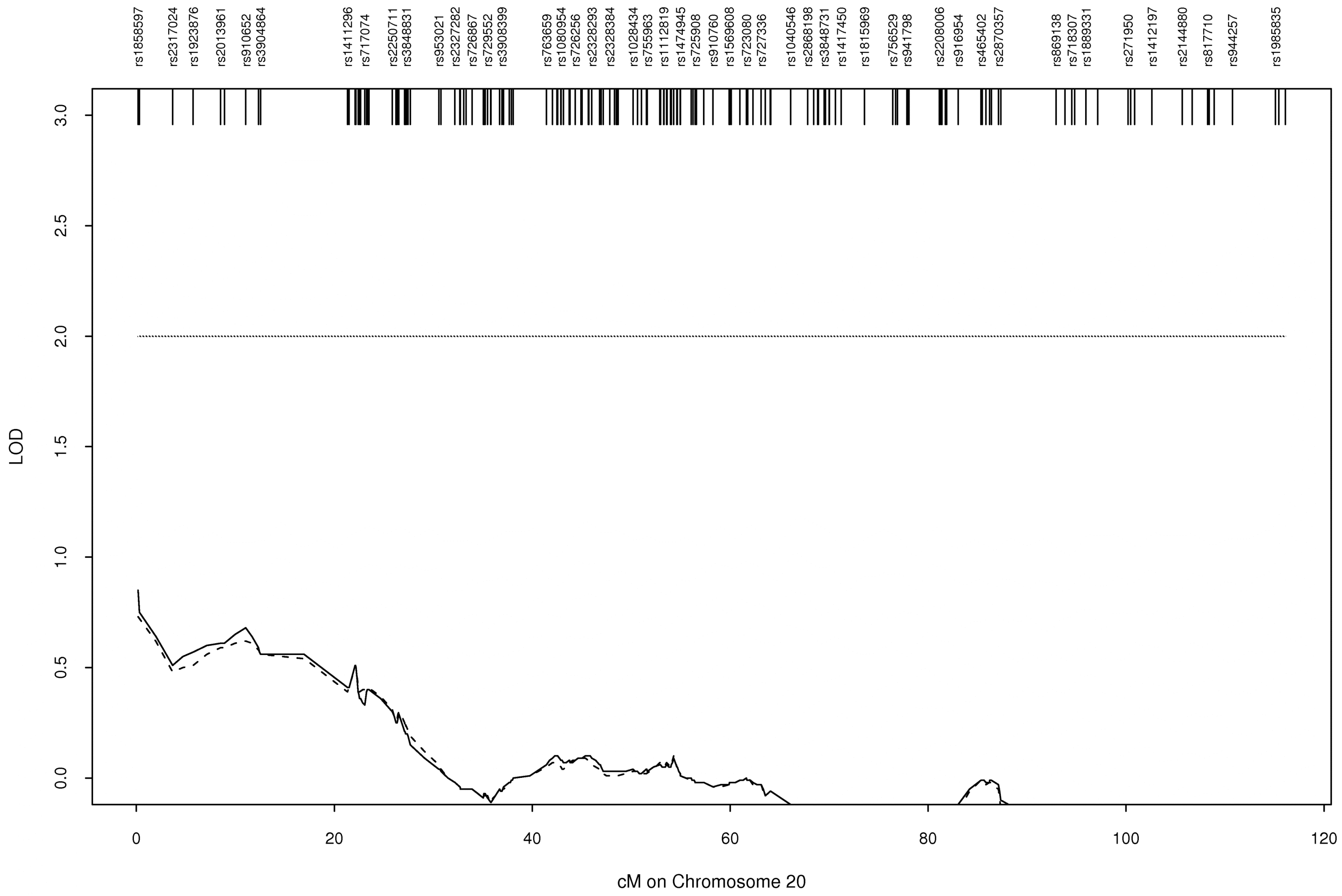

Casselbrant et al (2009) Otitis media: a genome-wide linkage scan with evidence of susceptibility loci within the 17q12 and 10q22.3 regions

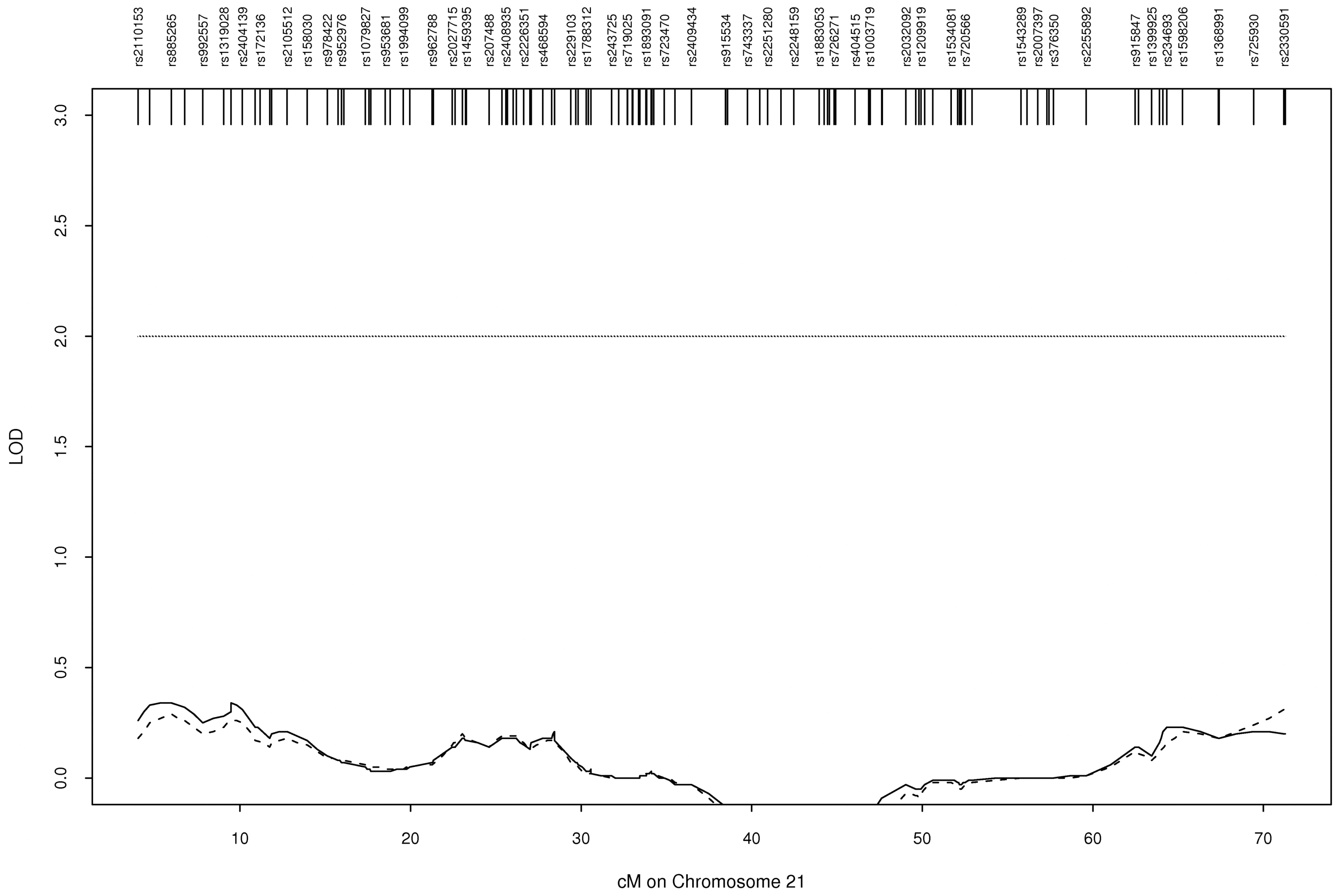

Casselbrant et al (2009) Otitis media: a genome-wide linkage scan with evidence of susceptibility loci within the 17q12 and 10q22.3 regions

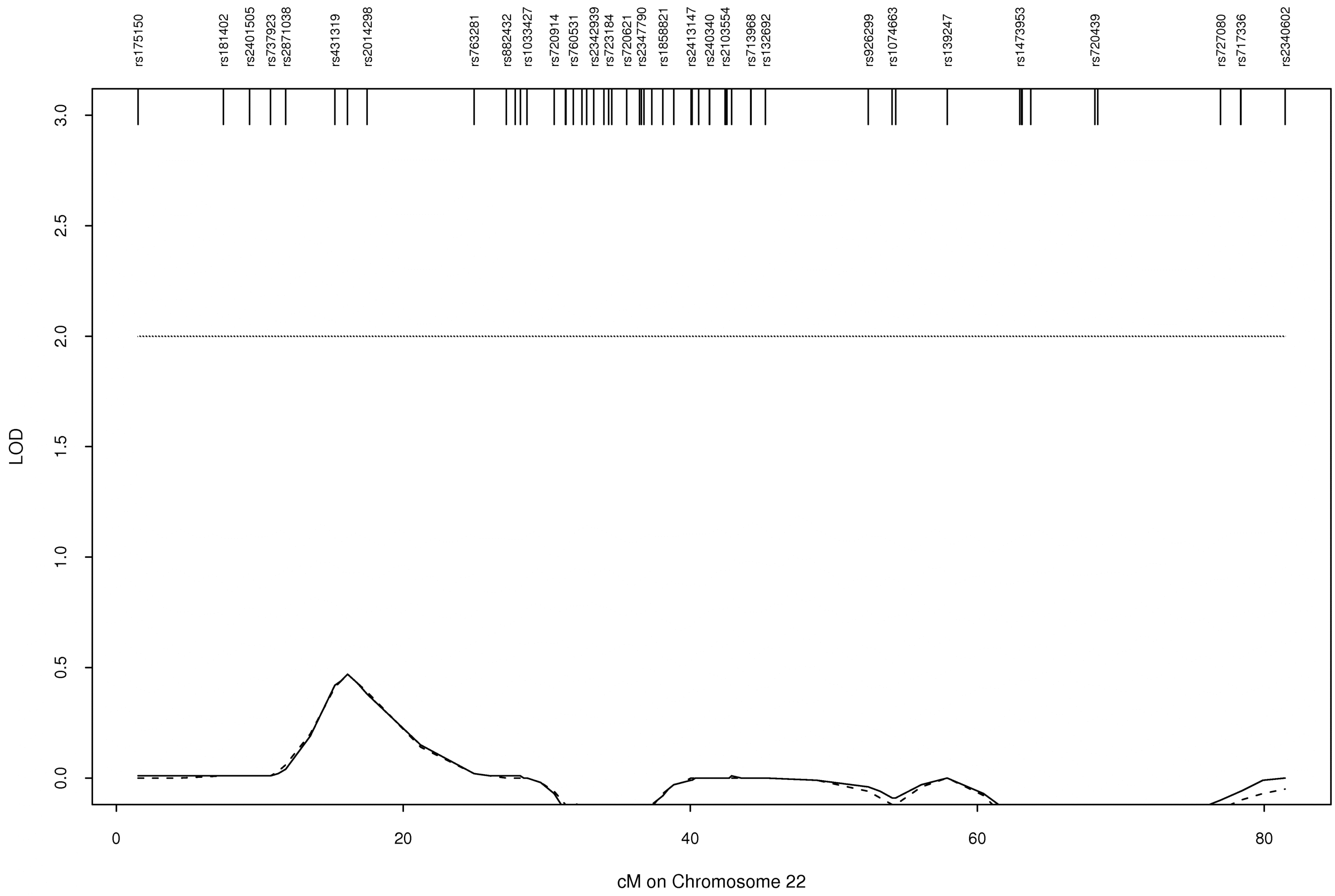

Casselbrant et al (2009) Otitis media: a genome-wide linkage scan with evidence of susceptibility loci within the 17q12 and 10q22.3 regions

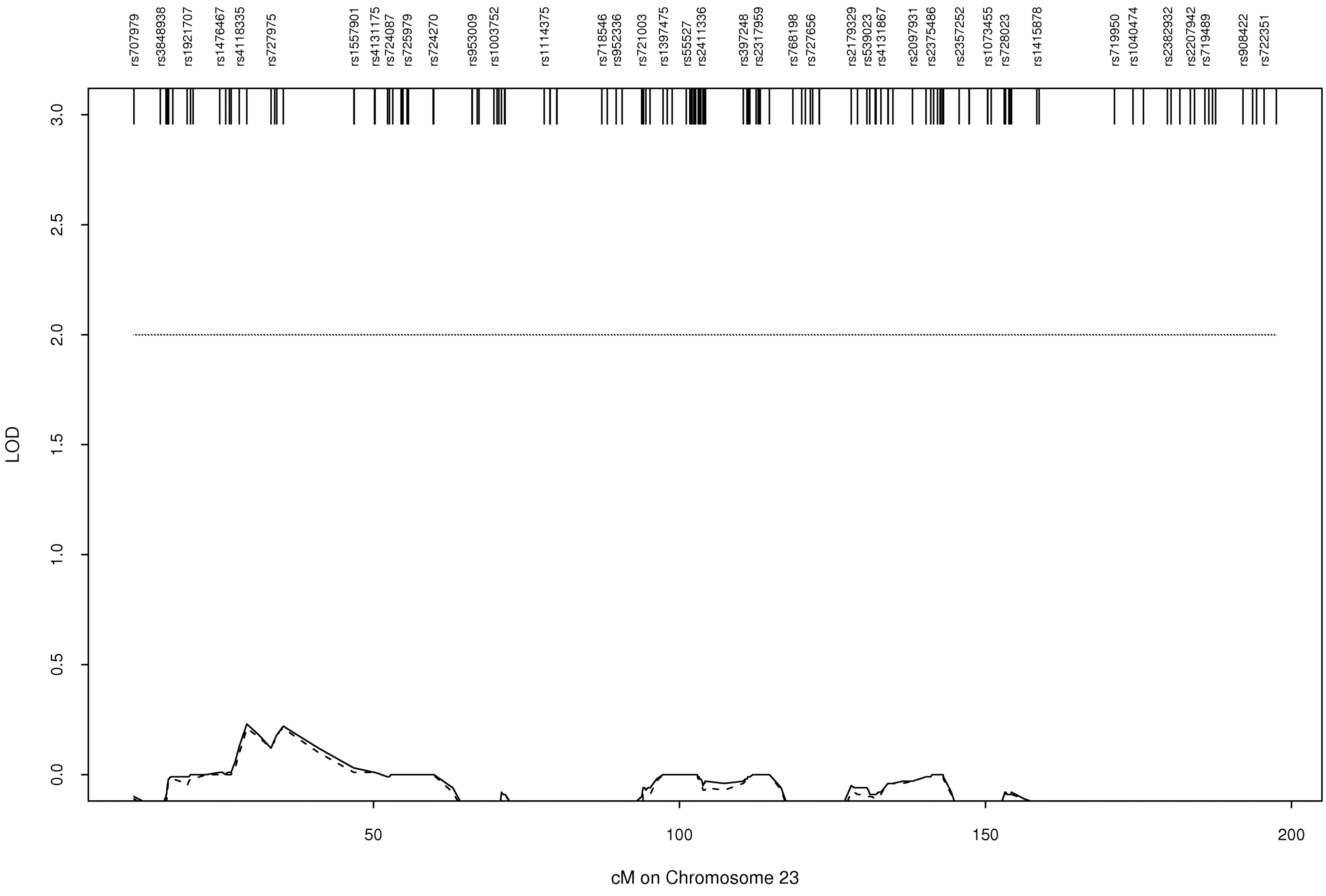

Supplement: Additional file 1 — Supplemental Figure S1. This file provides multipoint linkage curves as computed by Merlin with and without linkage disequilibrium. [file 1471-2350-10-85-S1.pdf]
