# Supplementary material for: Otitis media: a genome-wide linkage scan with evidence of susceptibility loci within the 17q12 and 10q22.3 regions
Source: BMC Med Genet. 2009 Sep 3;10:85. doi: 10.1186/1471-2350-10-85 (PMC2751750; doi:10.1186/1471-2350-10-85)
Supplement: Additional file 2 — Supplemental Table S1. This file provides the gamete competition results in the Caucasian families for those SNPs with p-values < 0.01. [file 1471-2350-10-85-S2.pdf]

Supplemental Table S1

Otitis media: a genome-wide linkage scan with evidence of susceptibility loci within the 17q12 and 10q22.3 regions

Margaretha L. Casselbrant, Ellen M. Mandel, Jeesun Jung, Robert E. Ferrell, Kathy Tekely, Jin P. Szatkiewicz, Amrita Ray, Daniel E. Weeks

Supplemental Table S1: SNPs with gamete competition p-values < 0.01 in the Caucasian families.

Rank	SNP	Chr.	Position	MAF	TransProb	P value	Closest gene	Distance to gene	Type
1	rs719898	4	169409966	0.05	0.34	6.6E-05	DDX60	0	Intronic
2	rs720758	11	36285306	0.08	0.37	8.2E-05	COMMD9	-17752	Intergenic
3	rs1000313	1	15278076	0.20	0.41	0.0002	RP1-21O18.1	0	Intronic
4	rs553288	3	167801369	0.17	0.41	0.0003	AC072046.11	69116	Intergenic
5	rs1437803	10	80472884	0.22	0.42	0.0005	ZMIZ1	-25914	Intergenic
6	rs1073712	1	64332677	0.27	0.42	0.0005	ROR1	0	Intronic
7	rs786074	1	82571608	0.41	0.43	0.0005	LPHN2	339301	Intergenic
8	rs788976	10	21228203	0.27	0.42	0.0006	NEBL	0	Intronic
9	rs1074449	9	4094571	0.32	0.43	0.0008	GLIS3	0	Intronic
10	rs1351650	11	7703386	0.34	0.36	0.0008	OR10AB1P	-2822	Upstream
11	rs722749	12	66786905	0.38	0.43	0.0009	IFNG	47911	Intergenic
12	rs2117032	12	20965389	0.38	0.43	0.0010	SLCO1B3	4464	Downstream
13	rs951937	3	163310228	0.22	0.36	0.0011			Intergenic
14	rs1398094	3	181028745	0.40	0.43	0.0012	PEX5L	0	Intronic
15	rs722748	12	66786791	0.39	0.44	0.0013	IFNG	48025	Intergenic
16	rs720616	7	124240197	0.39	0.43	0.0013	POT1	9480	Intergenic
17	rs1413724	6	86871268	0.32	0.43	0.0015	RP1-263J7.1	-17697	Intergenic
18	rs1904388	3	153229637	0.27	0.43	0.0015	SUCNR1	147611	Intergenic
19	rs181060	7	87947792	0.31	0.43	0.0016	AC002069.2	-31310	Intergenic
20	rs996724	1	82534635	0.42	0.44	0.0019	LPHN2	302328	Intergenic
21	rs1950245	14	39855383	0.29	0.43	0.0020			Intergenic
22	rs2213584	6	32521237	0.38	0.44	0.0021	HLA-DRA	436	Downstream
23	rs2227139	6	32521437	0.38	0.44	0.0021	HLA-DRA	636	Downstream
24	rs966536	11	42121577	0.29	0.38	0.0022			Intergenic
25	rs294665	2	123277166	0.17	0.42	0.0022			Intergenic
26	rs791856	6	86738560	0.32	0.43	0.0023	AL356601.14	20898	Intergenic
27	rs1645060	5	41427106	0.07	0.38	0.0024	PLCXD3	0	Intronic
28	rs978727	5	41338684	0.06	0.38	0.0026	PLCXD3	4121	Downstream
29	rs1208767	10	38082301	0.48	0.44	0.0029	RP11-162G10.4	40909	Intergenic
30	rs991408	8	54253908	0.44	0.44	0.0029	OPRK1	46921	Intergenic

31	rs1497250	12	25105913	0.10	0.32	0.0031	LRMP	0	Intronic
32	rs761801	6	40105154	0.32	0.43	0.0031	RP1-278E11.5	29204	Intergenic
33	rs922239	10	14165641	0.20	0.43	0.0032	FRMD4A	0	Intronic
34	rs1366151	5	162560591	0.13	0.42	0.0032	CCNG1	-236564	Intergenic
35	rs1415451	1	162392473	0.09	0.40	0.0033	RP11-541J2.1	-186393	Intergenic
36	rs1459865	11	21642888	0.49	0.44	0.0033	NELL1	89312	Intergenic
37	rs1388959	8	113540124	0.45	0.44	0.0034	CSMD3	0	Intronic
38	rs2250711	20	8203869	0.11	0.41	0.0034	PLCB1	0	Intronic
39	rs167164	2	123281397	0.18	0.43	0.0036			Intergenic
40	rs2067048	11	43116209	0.33	0.44	0.0036	API5	-173900	Intergenic
41	rs2892734	13	97815213	0.42	0.44	0.0038	FARP1	0	Intronic
42	rs2014048	21	15444217	0.08	0.40	0.0039	NRIP1	-85025	Intergenic
43	rs717482	16	84862498	0.26	0.44	0.0039	AC040170.9	4038	Downstream
44	rs1412996	13	26804257	0.23	0.43	0.0040	AL159977.10	11567	Intergenic
45	rs2368161	2	180869260	0.19	0.43	0.0042	CWC22	-289175	Intergenic
46	rs2373902	2	40744550	0.36	0.44	0.0044	SLC8A1	-52853	Intergenic
47	rs1987475	7	141837372	0.31	0.44	0.0047	U66060.1	-681	Upstream
48	rs150613	7	28583155	0.23	0.43	0.0048	CREB5	0	Intronic
49	rs2351463	5	138286701	0.32	0.44	0.0048	CTNNA1	0	Intronic
50	rs1751382	14	67762403	0.16	0.43	0.0050	RAD51L1	0	Intronic
51	rs504971	1	57337452	0.19	0.43	0.0051	DAB1	0	Intronic
52	rs2191039	7	13177325	0.29	0.44	0.0051	AC006000.4	-248255	Intergenic
53	rs1929715	9	82910738	0.45	0.44	0.0052	RP11-232A1.1	-304276	Intergenic
54	rs2017274	20	58902406	0.40	0.44	0.0052	AL117372.35	129425	Intergenic
55	rs42815	2	40679035	0.47	0.44	0.0053	SLC8A1	0	Intronic
56	rs1017003	7	8718368	0.33	0.44	0.0058	NXPH1	0	Intronic
57	rs1981497	14	105716737	0.07	0.40	0.0059	AB019440.1	-3695	Upstream
58	rs1931046	13	78270956	0.26	0.44	0.0062	RP11-600P1.3	37169	Intergenic
59	rs1074462	5	108970895	0.38	0.44	0.0063	AC114287.2	17815	Intergenic
60	rs708156	12	26583639	0.23	0.44	0.0064	ITPR2	0	Intronic
61	rs728695	9	84651341	0.35	0.44	0.0065	RP11-22C13.1	-101814	Intergenic
62	rs1410281	13	25798920	0.07	0.39	0.0065	CDK8	0	Intronic
63	rs411280	11	130897898	0.35	0.44	0.0066	HNT	0	Intronic
64	rs2325123	13	67756047	0.37	0.44	0.0066	RP11-520F24.2	5765	Intergenic

65	rs718878	6	163723618	0.17	0.43	0.0073	RP3-495O10.1	19133	Intergenic
66	rs2161662	16	59401492	0.12	0.42	0.0074	AC009156.10	105399	Intergenic
67	rs154948	5	64906889	0.29	0.44	0.0074	PPWD1	0	Intronic
68	rs1944932	11	110807220	0.43	0.45	0.0077	BTG4	36245	Intergenic
69	rs1121682	10	34532194	0.26	0.44	0.0077	PARD3	0	Intronic
70	rs764682	16	8423860	0.36	0.45	0.0077	C16orf68	-199168	Intergenic
71	rs892458	2	34521253	0.43	0.40	0.0078	AC011748.7	38925	Intergenic
72	rs953932	3	41542966	0.16	0.43	0.0079	ULK4	0	Intronic
73	rs1427202	10	59937923	0.50	0.45	0.0082	BICC1	-4987	Upstream
74	rs1073936	16	64272020	0.26	0.44	0.0086			Intergenic
75	rs717267	9	16398826	0.39	0.45	0.0087	BNC2	675	Downstream
76	rs1113990	8	28758718	0.36	0.45	0.0087	INTS9	0	Intronic
77	rs950110	4	73054396	0.35	0.45	0.0088	NPFFR2	-61989	Intergenic
78	rs717821	3	60490818	0.07	0.40	0.0088	FHIT	0	Intronic
79	rs953163	3	68967916	0.46	0.45	0.0089	FAM19A4	0	Intronic
80	rs1995688	6	86689038	0.32	0.44	0.0092	RP11-207F8.1	53137	Intergenic
81	rs717453	10	59938404	0.49	0.45	0.0092	BICC1	-4506	Upstream
82	rs1022109	3	68578614	0.30	0.45	0.0093	FAM19A1	0	Intronic
83	rs717452	10	59938276	0.49	0.45	0.0095	BICC1	-4634	Upstream
84	rs788975	10	21227958	0.09	0.41	0.0095	NEBL	0	Intronic
85	rs727998	6	93802155	0.33	0.45	0.0096	AL138731.10	56260	Intergenic
86	rs925939	8	139603308	0.17	0.37	0.0097	FAM135B	-25061	Intergenic
87	rs1938887	11	99767680	0.23	0.44	0.0097	CNTN5	34997	Intergenic
88	rs1588908	3	162871507	0.47	0.45	0.0098	AC104471.6	167083	Intergenic

MAF = estimated minor allele frequency; TransProb = probability of transmission from a heterozygous genotype; Distance to gene is given in base pairs.
